# Inhibition of LSD1 induces ferroptosis through the ATF4-xCT pathway and shows enhanced anti-tumor effects with ferroptosis inducers in NSCLC

**DOI:** 10.1038/s41419-023-06238-5

**Published:** 2023-11-03

**Authors:** Linna Du, Han Yang, Yufei Ren, Yanli Ding, Yichao Xu, Xiaolin Zi, Hongmin Liu, Pengxing He

**Affiliations:** 1 School of Pharmaceutical Sciences, Zhengzhou University, Zhengzhou, 450001 China; 2 Departments of Urology and Pharmaceutical Sciences and Chao Family Comprehensive Cancer Center, University of California, Irvine, Irvine, CA 92697 USA

**Keywords:** Cancer therapy, Non-small-cell lung cancer, Cell death

## Abstract

Lysine-specific demethylase 1 (LSD1) has been identified as an important epigenetic target, and recent advances in lung cancer therapy have highlighted the importance of targeting ferroptosis. However, the precise mechanisms by which LSD1 regulates ferroptosis remain elusive. In this study, we report that the inhibition of LSD1 induces ferroptosis by enhancing lipid peroxidation and reactive oxygen species (ROS) accumulation. Mechanistically, LSD1 inhibition downregulates the expression of activating transcription factor 4 (ATF4) through epigenetic modification of histone H3 lysine 9 dimethyl (H3K9me2), which sequentially inhibits the expression of the cystine–glutamate antiporter (xCT) and decreases glutathione (GSH) production. Furthermore, LSD1 inhibition transcriptionally upregulates the expression of transferrin receptor (TFRC) and acyl-CoA synthetase long chain family member 4 (ACSL4) by enhancing the binding of histone H3 lysine 4 dimethyl (H3K4me2) to their promoter sequences. Importantly, the combination of an LSD1 inhibitor and a ferroptosis inducer demonstrates an enhanced anti-tumor effect in a xenograft model of non-small cell lung cancer (NSCLC), surpassing the efficacy of either agent alone. These findings reveal new insights into the mechanisms by which LSD1 inhibition induces ferroptosis, offering potential guidance for the development of new strategies in the treatment of NSCLC.

## Introduction

Ferroptosis is a unique iron-dependent form of non-apoptotic cell death characterized by the excessive accumulation of lipid hydroperoxides and reactive oxygen species (ROS) [1]. The cystine-glutamate transporter system X_C_^−^, a heterodimer composed of xCT (also named SLC7A11, encoded by *SLC7A11*) and SLC3A2 (also named 4F2hc, encoded by *SLC3A2*), accounts for the transportation of extracellular cystine into cells for GSH synthesis [2–5]. Glutathione peroxidase 4 (GPX4) is the sole member of the GSH peroxidase family that can efficiently reduce uncontrolled peroxidation of phospholipids and cholesterol to their corresponding alcohols [6, 7]. Acyl-CoA synthetase long-chain family member 4 (ACSL4) is a key enzyme that facilitates the esterification of arachidonoyl and adrenoyl into phosphatidylethanolamine in cellular membranes, which has been shown to contribute to the execution of ferroptosis. Pharmacological targeting of System X_C_^−^, GPX4, and ACSL4 with erastin (ERA), (1 S, 3 R)-RSL3 (RSL) and thiazolidinediones induces ferroptosis [8–11]. In fact, ferroptosis has recently become an important cancer target and ferroptosis inducers are being rapidly developed to treat cancer in recent years [12]. In particular, ferroptosis inducer ERA and its combination with the nuclear factor erythroid 2–related factor 2 (NRF2) inhibitor (brusatol) have been proved to be useful for the treatment of non-small cell lung cancer (NSCLC) [13, 14].

Recent studies have found that many epigenetic regulators are involved in regulating ferroptosis. Histone lysine demethylase 3B (KDM3B), a histone H3 lysine 9 demethylase, was shown to upregulate the expression of xCT and confer resistance to ERA [15]. Another study found that bromodomain-containing protein 4 (BRD4) controls the expression of ferroptosis-associated proteins including GPX4, xCT, and SCL3A2 through its interaction with transcription factors [16]. In addition, H2A deubiquitination but H2B ubiquitination on the xCT promoter increases the expression of xCT, thereby inhibiting lipid peroxidation and ferroptosis [17]. Lysine-specific demethylase 1 (LSD1) has fundamental roles in the development of various pathological conditions involving cell proliferation [18], cell cycle progression [19], autophagy [20], DNA damage [21], and worse prognosis of cancer patients [22]. Thus, LSD1 has been recognized as an important epigenetic target for improving cancer therapy [23, 24]. We have previously shown that LSD1 is overexpressed in NSCLC and confirmed that Inhibition of LSD1 reduced tumor growth and metastasis [25]. Very recently Lu et al have reported that suppression of LSD1 expression by shRNA increased the intracellular malondialdehyde (MDA) and Fe^2+^ levels and enhanced ERA and RSL-induced ferroptosis in human lung cancer cells [26]. However, the mechanism by which LSD1 regulates ferroptosis remains largely unknown.

In this study, we have shown that inhibition of LSD1 repressed activating transcription factor 4 (ATF4) expression, which subsequently inhibited xCT expression, resulting in reduced GSH production and ferroptosis. In addition, inhibiting LSD1 upregulated the expression of transferrin receptor 1 (TFRC) and ACSL4. Moreover, combination of LSD1 inhibitors and the ferroptosis inducer RSL resulted in an enhanced inhibition of tumor growth in a xenograft model of human lung cancer cells compared to either treatment alone.

## Materials and methods

### Reagents and antibodies

ORY-1001 (ORY; HY-12782T), Erastin (ERA; HY-15763), (1S,3R)-RSL3 (RSL; HY-100218A), Ferrostatin-1 (Fer-1; HY-100579), Z-VAD-FMK (ZVAD; HY-16658B), Bafilomycin A1 (BA1; HY-100558), and Necrostatin-1 (Nec1; HY-15760) were purchased from MedChemExpress (China). GSK-LSD1 (GSK; S7574) was purchased from Selleck Chemicals (China). GSK-2879552 was obtained from Meilunbio (China). The primary antibodies used here include: GAPDH (Immunoway, YT5052), β-actin (Sungene biotech, KM9001T), ACSL4 (Invitrogen, PA527137), TFRC (Abcam, ab214039), GPX4 (Abcam, ab125066), xCT (Abcam, ab37185), ATF4 (Cell Signaling Technology, 11815), LSD1 (Cell Signaling Technology, 2184), H3K9me2 (Cell Signaling Technology, 4658), YAP (Cell Signaling Technology, 14074), p-YAP (Cell Signaling Technology, 13008), FPN (Abcam, ab1239583), DMT1 (Abcam, ab55735), Ferritin (Abcam, 287968), p-STAT1 (Cell Signaling Technology, 9167), STAT1 (Cell Signaling Technology, 9172), PARP (Cell Signaling Technology, 9542), and LC3B (Cell Signaling Technology, 3868). HRP-linked anti-rabbit IgG (Jackson, 111-035-003, 1:5,000 dilution, USA) and HRP-linked anti-mouse IgG (Jackson, 615-635-214, 1:5,000 dilution, USA) were used as the secondary antibodies.

### Cell culture

Lung cancer cell lines (H1299, PC9, A549, H1650, H460, and H1975) and normal lung epithelial cells (BEAS-2B) (Supplementary Table 3) were cultured in RPMI 1640 (Biological Industries, Israel) medium supplemented with 10% FBS (Biological Industries, Israel). All cells were incubated at 37 °C in an incubator containing 5% CO_2_.

### Cell viability assays

The effects of compounds on lung cancer cells were analyzed by sulforhodamine B (SRB; Sigma, USA) assays. Briefly, cells were plated in 96-well plates and cultivated in an incubator (37 °C, 5% CO_2_) for the night, then treated with compounds for 96 h. Cells were fixed with 10% trichloroacetic acid, washed three times with distilled water and then stained with SRB in 1% acetic acid. SRB present in the cells was dissolved in 10 mM Trise-HCl. Finally, the absorbance at 560 nm was measured with a multimode microplate reader (PerkinElmer, USA).

### Western blot analysis

Western blotting was performed as previously described according to the standard protocol [27, 28]. Briefly, treated cells were lysed with RIPA buffer (Solarbio, China) with a protease inhibitor cocktail. Equal amounts of proteins were resolved by SDS‒PAGE and transferred to nitrocellulose membranes (Thermo Scientific, USA). Membranes were probed with the primary antibodies and then further incubated with secondary antibodies and visualized with ECL (Thermo Scientific).

### The reactive oxygen species assay

ROS levels were measured by a ROS Assay Kit (Beyotime Biotechnology, S0033S, China) which is a kind of reactive oxygen species detection kit with fluorescent probe DCFH-DA [29]. To visualize the ROS production, cells were stained with 10 μM DCFH-DA for 30 min in the dark at 37 °C. The cells were washed with PBS and the fluorescence intensity of DCF was measured via flow cytometry (FACSCalibur, BD Biosciences, USA).

### Lipid peroxides assay

The lipid peroxides levels were measured using an oxidation-sensitive fluorescent lipid peroxidation probe C11-BODIPY581/591(Invitrogen, D3861). Briefly, cells were stained with 5 μM C11-BODIPY581/591 for 30 min at 37 °C. Subsequently, cells were washed with PBS and detected by flow cytometric analysis.

### Cell death assay

To visualize the cell death, cells were seeded in six-well plates with 40,000 cells/well and treated with the designated conditions. Then, cells were harvested by trypsinization and were stained with SYTOX™ Green Dead Cell Stain (Thermo Scientific, S34860) for 30 min at 37 °C. After washing with PBS, cells were detected by FACS analysis.

### GSH assay

Cellular GSH levels were measured using the Total Glutathione Assay Kit (Beyotime Biotechnology, S0052). Briefly, cells were washed with PBS and protein removal reagent S solution was added. Later, the cells were quickly lysed via freeze/thawing twice and were centrifuged at 10,000×g for 10 min at 4 °C according to the manufacturer’s instructions. The supernatant was mixed with 2 mM 5,5′-dithio-bis (2-nitrobenzoic acid), Glutathione reductase, and NADPH. The GSH levels were measured via the glutathione reductase recycle assay. The optical density at 412 nm was read using a multimode microplate reader (PerkinElmer, USA) and the GSH concentrations were calculated against a standard curve.

### Plasmids transfection

Reprogramming plasmids pRK-ATF4 (#26114), plenti-ATF4 (#125238), plenti6-xCT-V5 (#170427), pDONR221-xCT (#132244), and pFETCh_KDM1A (#86260) were obtained from Addgene (USA) as bacterial stabs. These plasmids were transfected using Entranster-H4000 (Engreen Biosystem, China). Proteins were extracted 48 h after transfection to detect the expression levels of related proteins.

### siRNA transfection

The siRNA sequence was synthesized by Shanghai GenePharma (China). The sequences were designed as follow: siLSD1 (sense 5′-CACAAGGAAAGCUAGAAGATT-3′), sixCT#1 (sense 5′-CCAGGUGGUUUAGAAUAAUTT-3′), sixCT#2 (sense 5′-CCAGAUAUGCAUCGUCCUUTT-3′), siACSL4 (sense 5′-GAGGCUUCCUAUCUGAUUATT-3′), and siTFRC (sense 5′-ACAAGUUAGAGAAUGCUGAUCUGGC-3′). The irrelevant nucleotides not targeting any annotated human genes were used as negative control: siNC (sense 5′-UUCUCCGAACGUGUCACGUTT-3′). These siRNAs were transfected using Entranster-H4000 (Engreen Biosystem, China). Proteins were extracted 48 h after transfection to detect the expression levels of related proteins.

### CRISPR/Cas9 knockout

A dual sgRNA-directed CRISPR/Cas9 system was used to generate LSD1-specific knockout cells. The dual sgRNA was synthesized by Genewiz (Suzhou, China) and subcloned into the Cas9/gRNA vector (Viewsolid Biotech, Beijing) to yield their corresponding CRISPR/gRNA expression vectors: LSD1-sgRNA. The lentivirus for knocking out LSD1 were produced by co-transfection of 293 T cells with packaging plasmid, envelope plasmid, and LSD1-sgRNA using FuGENE HD in accordance with the manufacturer’s protocol. Virus was harvested after 48 h, filtered, and used to infect A549 cells in the presence of 5 μg/mL polybrene via spin infection (2500 rpm, 30 min). Selection of resistant colonies was initiated 24 h later using 2 μg/mL puromycin.

### Chromatin immunoprecipitation and quantitative RT-PCR

Chromatin immunoprecipitation (ChIP) was performed by a SimpleChIP® Plus Enzymatic Chromatin IP Kit (Magnetic Beads, Cell Signaling Technology, 9005 S). Briefly, cells were crosslinked with 1% formaldehyde in PBS, quenched with 2.5 M glycine and washed with PBS three times. Nuclei were prepared, and chromatin was incubated with micrococcal nuclease at 37 °C for 20 min. The supernatants were immunoprecipitated by being incubated with 10 µl of anti-H3K9me2 or nonspecific rabbit IgG at 4 °C for 12–16 h. The immunocomplexes were rotationally incubated with 30 µl of ChIP-Grade Protein G Magnetic Beads for 2 h at 4 °C and then were washed three times using low salt wash buffer and 1 time with high salt wash buffer at 4 °C. Chromatin was eluted by ChIP elution buffer for 30 min at 65 °C and crosslinks were reversed by treatment with 5 M NaCl and proteinase K overnight at 65 °C. Samples were then incubated with RNase at 37 °C for 1 h. CHIP DNA was purified and subsequently quantified by quantitative real-time PCR (qRT-PCR). Quantitative ChIP confirmed changes at the promoters of examined genes using qPCR with primer sets indicated in Supplementary Table 1. Data analysis was finally presented as percentages of the input DNA. The sequences of the primers used for the qPCR were listed in Supplementary Table 2.

### MDA assay

The tumor tissue proteins were normalized according to their concentrations and subjected to a malondialdehyde (MDA) assay according to the instructions provided by the Lipid Peroxidation MDA assay kit (Beyotime Biotechnology, S0131S). The MDA levels were determined using a multimode microplate reader (PerkinElmer, USA) at 532 nm.

### Animal experiments

Athymic BALB/c 4–6 weeks old nude mice were purchased from Hunan Slack Scene of Laboratory Animal Company Ltd. (Hunan, China) and were housed in a specific pathogen-free conditions. All animal experiments were performed according to the institutional ethical guidelines established by the ethics committee of Zhengzhou University (China). The A549 cells at a density of 4 × 10^6^ in 200 μL were subcutaneously implanted into the right flank of the nude mice. Once the tumor volume reached 100–200 mm^3^, mice were randomly divided into control and treatment groups (*n* = 5 per group): (1) control group, ig; (2) ORY (400 μg/kg/7days), ig; (3) RSL (100 mg/kg/4days/), ip; (4) ORY + RSL. Tumor-bearing mice were treated with drugs for four weeks. The sizes of the tumors were measured with a microcaliper every 2 days until the endpoint and the tumor volume was calculated according to the equation volume =1/2(Length × Width^2^).

### Statistical analysis

All data represent at least 4 independent experiments and are expressed as mean ± standard deviation (s.d.). Statistical significance between the groups was determined using One-way ANOVA. *n* ≥ 3; mean ± SEM; *P < 0.05; **P < 0.01; ***P < 0.001.

## Results

### Inhibition of LSD1 induces ferroptosis

**Fig. 1:**
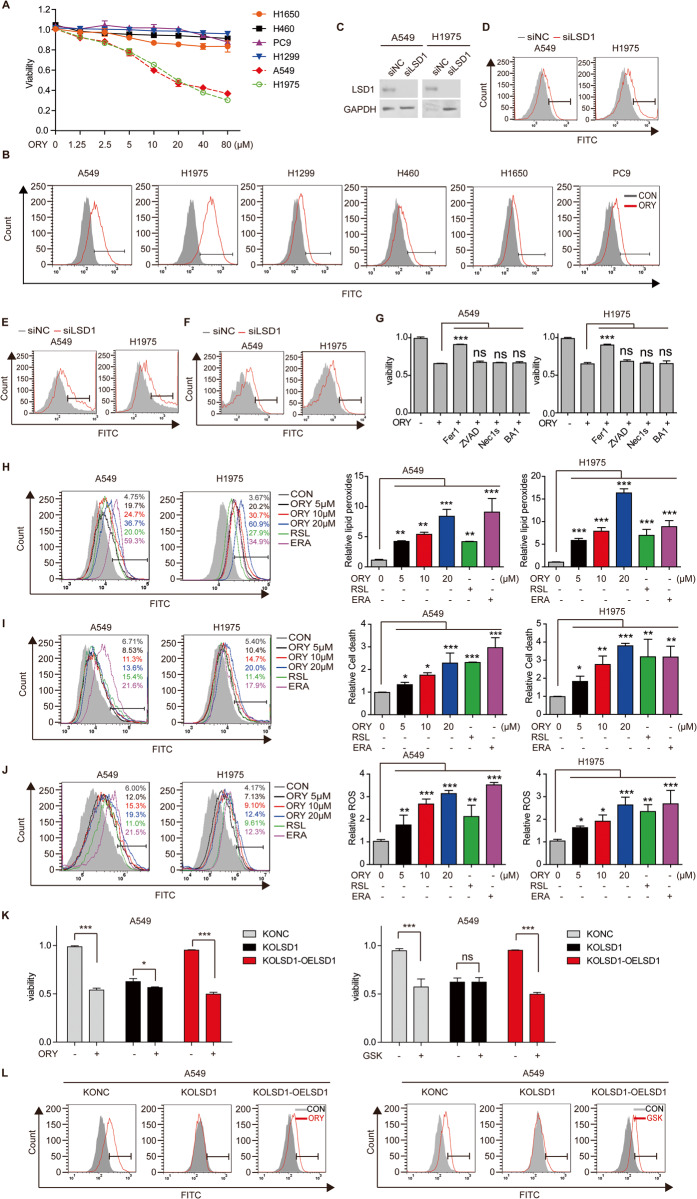
Inhibition of LSD1 induces ferroptosis. **A** Cell viability was detected by SRB assay in NSCLC cell lines treated with ORY at increasing concentrations for 96 h. **B** Lipid peroxides were measured by flow cytometry in NSCLC cells treated with 10 μM ORY for 96 h. **C**–**F** After A549 and H1975 cells were transfected with LSD1 siRNA for 48 h, LSD1 expression (**C**) was analyzed by Western blotting, Lipid peroxides (**D**), cell death (**E**), and total ROS accumulation (**F**) were measured by flow cytometry. **G** After NSCLC cell lines were treated with 10 μM ORY for 48 h and 5 μM Fer-1, 5 μM ZVAD, 2 μM Nec1, and 10 nM BA1 were added again for 48 h as indicated, cell viability was measured by SRB assay. **H**–**J** After A549 and H1975 cells were treated with 5 μM, 10 μM or 20 μM ORY for 96 h, 20 μM ERA for 48 h or 2 μM RSL for 48 h as indicated, lipid peroxides (**H**), cell death (**I**), and total ROS accumulation (**J**) were analyzed by flow cytometry. **K**, **L** After A549-KOLSD1 cell lines were transfected with pIRES2-EGFP-LSD1 plasmid and were treated with 10 μM ORY for 96 h, cell viability (**K**) was detected by SRB, and production of lipid peroxides (**L**) was analyzed by flow cytometry. *n* ≥ 3; Mean ± SEM; ^ns^*P* > 0.05; *P < 0.05; **P < 0.01; ***P < 0.001.

To investigate the role of LSD1 in ferroptosis in a panel of NSCLC cell lines and normal lung epithelial cells, LSD1 reversible inhibitors ORY and GSK were used. Most tested cell lines showed resistance to LSD1 inhibitors, but A549 and H1975 cells were consistently sensitive (Fig. 1A; Supplementary Fig. 1A; and Supplementary Fig. 2A). We found that the LSD1 inhibitor-sensitive cell lines had significantly elevated levels of lipid peroxidation when treated with ORY (Fig. 1B and Supplementary Fig. 2B). Cell death was stained using SYTOX Green staining, which indicates ferroptosis [30], and ROS accumulation is closely related to ferroptosis [1]. Consistently, knocking down LSD1 resulted in lipid peroxidation, cell death and ROS accumulation, indicating that cells undergo ferroptosis (Fig. 1C-F). Although previous studies have shown that LSD1 inhibitors can induce both apoptosis [31] and autophagy [20], our results showed that ORY significantly induced autophagy but not apoptosis (Supplementary Fig. 1B, C). To explore the contribution of different programmed cell deaths to LSD1 inhibition, ferroptosis inhibitor Fer-1, apoptosis inhibitor ZVAD, necroptosis inhibitor Nec1, and the autophagy inhibitor BA1 were utilized. The results showed that ORY and GSK-mediated responses were specifically blocked by Fer-1 rather than other inhibitors (Fig. 1G and Supplementary Fig. 1D), suggesting that the effectiveness of LSD1 inhibitor to induce apoptosis and autophagy is limited in this study. Thus, inhibiting LSD1 induces ferroptosis in A549 and H1975 cells.

We compared the lipid peroxidation, cell death, and ROS levels induced by ORY, GSK, and ferroptosis inducers ERA and RSL. Consistent with ferroptosis inducers, ORY and GSK induced lipid peroxidation, cell death, and ROS in a concentration-dependent manner (Fig. 1H-J and Supplementary Fig. 1E–G). Therefore, inhibiting LSD1 drives ferroptosis by promoting ROS accumulation and lipid peroxidation. To further validate the role of LSD1 in the regulation of ferroptosis, LSD1 expression was knocked out in A549 cell lines (A549-KOLSD1) using the CRISPR/Cas9 system. Subsequently, we transferred the pIRES2-EGFP-LSD1 plasmid to restore LSD1 expression in A549-KOLSD1 cell line (A549-KOLSD1-OELSD1) (Supplementary Fig. 1L). The findings revealed that ORY or GSK exhibited minimal effects on cell viability and lipid ROS levels in the KOLSD1 cell line. However, the ability of LSD1 inhibitors to induce ROS elevation and to reduce cell viability was reinstated in the KOLSD cell line with re-expression of LSD1 (Fig. 1K, L). Simultaneously, we performed a knockdown of LSD1 to investigate the targeting characteristics of LSD1 inhibitors. The results demonstrated that following the transfection of siLSD1, LSD1 inhibitors did not result in a decrease in cell viability and an increase in lipid ROS (Supplementary Fig. 1H, I, K). In order to further investigate the impact of LSD1 inhibition on different programmed cell death pathways, Fer-1, ZVAD, Nec1, and BA1 were used. The results revealed that the effects mediated by siLSD1 were specifically counteracted by Fer-1, rather than the other inhibitors (Supplementary Fig. 1J). This finding suggests that the cell death triggered by LSD1 knockdown is primarily attributed to ferroptosis. In addition, A549-KOLSD1 cells with restoration of LSD1 expression were exposed to ORY/GSK, Fer-1, or a combination of both, and their effects on cell viabilities were assessed. Our results demonstrated that Fer-1 effectively restored the reduced cell viability caused by ORY/GSK treatment in A549-KOLSD1-OELSD1 cells (Supplementary Fig. 1M). These results suggest that LSD1 expression is required, at least in part, for the LSD1 inhibitors-induced ferroptosis in A549 and H1975 cells.

### Elevated intracellular iron is required for ferroptosis induction by LSD1 inhibitors

**Fig. 2:**
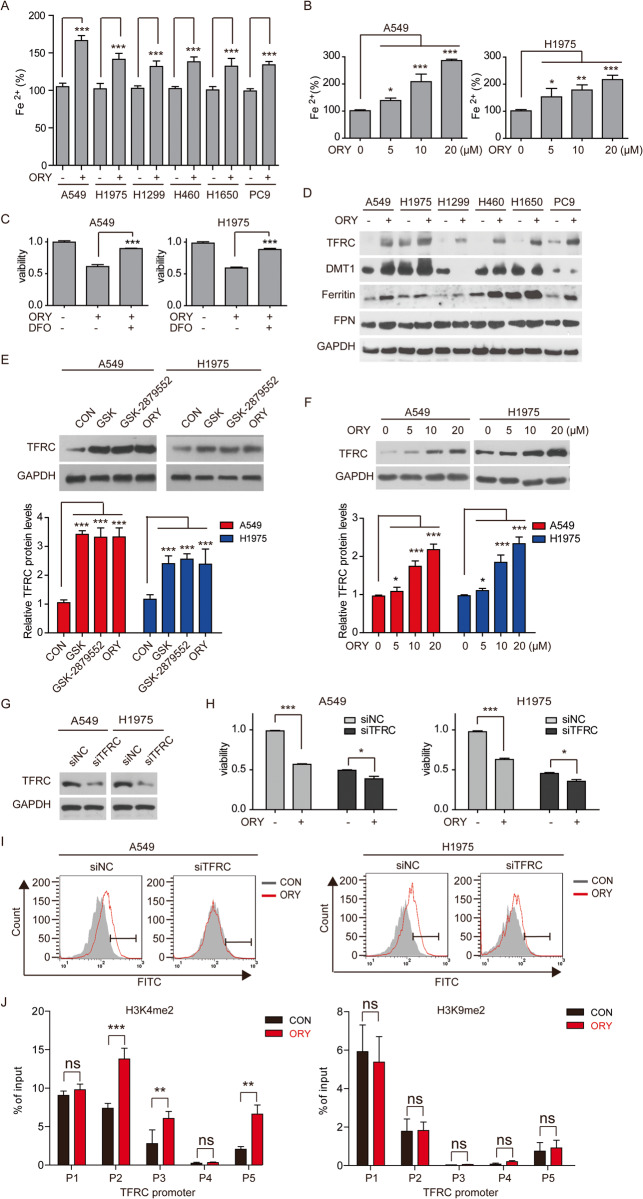
Inhibiting LSD1 regulates intracellular iron overload through iron-regulating proteins. **A** The content of Fe^2+^ was measured in NSCLC cells treated with 10 μM ORY for 96 h. **B** Fe2+ levels were determined in A549 and H1975 cell lines treated with gradient concentrations of ORY for 96 h. **C** After NSCLC cell lines were treatment with 10 μM ORY for 48 h and 10 μM DFO was added again for 48 h as indicated, cell viability was measured by SRB. **D** The expression of TFRC, DMT1, ferritin and FPN was examined by western blotting in NSCLC cells treatment with 10 μM ORY for 96 h. **E** TFRC expression was determined by western blotting in A549 and H1975 cell lines treated with 10 μM GSK, 10 μM GSK-2879552 or 10 μM ORY for 96 h as indicated. **F** TFRC expression was determined by western blotting in A549 and H1975 cell lines treated with increasing concentrations of ORY for 96 h. **G**–**I** After A549 and H1975 cell lines were transfected with TFRC siRNA and were treated with 10 μM ORY for 96 h, TFRC expression (**G**) was determined by western blotting, cell viability (**H**) was detected by SRB, and production of lipid peroxides (**I**) was analyzed by flow cytometry. **J** Quantitative ChIP studies were conducted to characterize the enrichment of H3K4me2 and H3K9me2 at promoters of *TFRC* gene in A549 cells treated with 10 μM ORY for 96 h. *n* ≥ 3; Mean ± SEM; *P < 0.05; **P < 0.01; ***P < 0.001.

Considering that iron plays important role in ferroptosis through Fenton reaction [32], we measured the levels of Fe^2+^ in cells. The results showed that ORY treatment led to the accumulation of Fe^2+^ in all tested NSCLC cells (Fig. 2A, B). Importantly, the iron chelator deferoxamine (DFO) abolished ORY-induced tumor cell death in sensitive NSCLC cells (Fig. 2C). The intracellular iron level is regulated by its uptake, storage, release, and metabolism [33]. Fe^3+^ is taken up by TFRC into the endosome, where it is reduced to Fe^2+^ by STEAP3 and then released into the cytoplasm by DMT1 [34, 35]. Ferritin is the primary site of iron storage in the cell and serves as a protective agent against oxidative stress [36]. Ferroportin (FPN, also called Scl40a1), the sole known mammalian iron exporter [37], releases Fe^2+^ into the extracellular space and plays a key role in balancing cellular iron levels [38]. Among the key genes involved in iron metabolism (e.g., TFRC, DMT1, ferritin, and FPN), the upregulation of TFRC and ferritin was apparent in all tested NSCLC cell lines upon ORY treatment (Fig. 2D). Consistently, mRNA and protein levels of TFRC was increased by a variety of LSD1 inhibitors (Fig. 2E, F and Supplementary Fig. 3A, B). In line with this observation, the knockdown of LSD1 led to an increase in TFRC mRNA levels (Supplementary Fig. 3C). In addition, knockdown of TFRC suppressed ORY and GSK-induced cell death and lipid peroxides production (Fig. 2G–I and Supplementary Fig. 3D, E). Thus, these results demonstrate that TFRC is required for ferroptosis induced by LSD1 inhibitors in sensitive cell lines. ChIP-qPCR showed that inhibition of LSD1 increased H3K4me2 levels, but not H3K9me2 levels, at TFRC promoter (Fig. 2J), suggesting that LSD1 inhibitor promoted TFRC transcription via H3K4me2.

### ACSL4 is involved in LSD1 inhibitor-induced ferroptosis

**Fig. 3:**
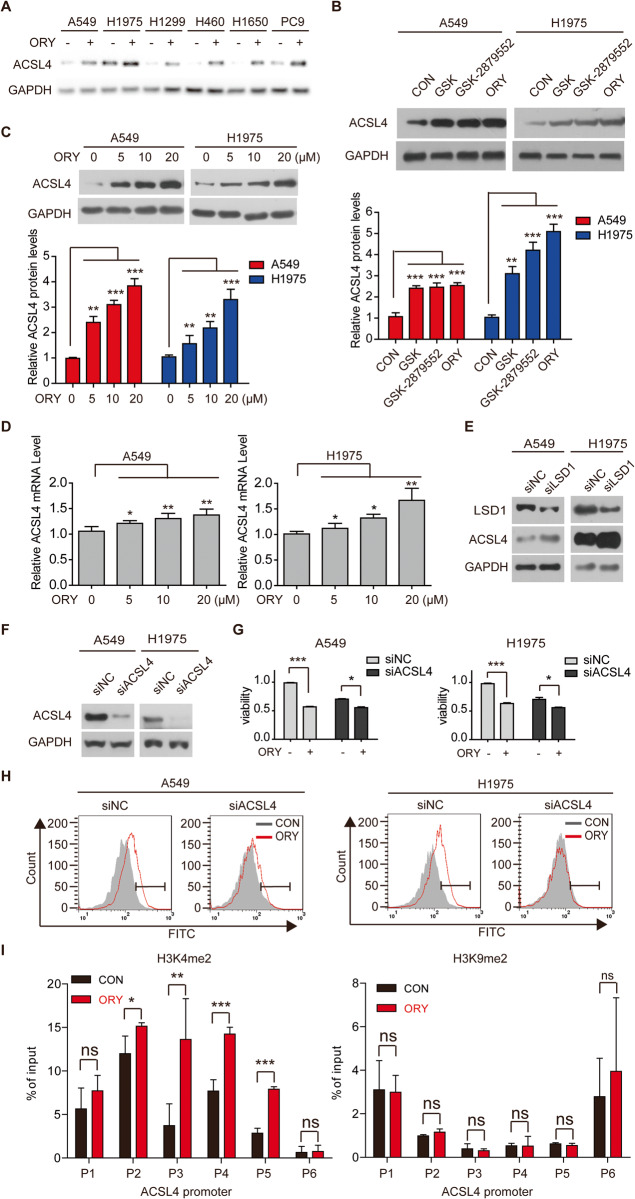
Upregulating ACSL4 is involved in LSD1 inhibitor-induced ferroptosis. **A** The expression of ACSL4 was examined in NSCLC cells treatment with 10 μM ORY for 96 h. **B** ACSL4 expression was determined by western blotting in A549 and H1975 cell lines treated with 10 μM GSK, 10 μM GSK-2879552, or 10 μM ORY for 96 h as indicated. **C** ACSL4 expression was determined by western blotting in A549 and H1975 cell lines treated with increasing concentrations of ORY for 96 h. **D** ACSL4 mRNA was determined by qRT-PCR in A549 and H1975 cell lines treated with increasing concentrations of ORY for 96 h. **E** The expression of LSD1 and ACSL4 was analyzed by western blotting in A549 and H1975 cell lines transfected with ACSL4 siRNA for 48 h. **F**–**H** After A549 and H1975 cell lines transfected with ACLS4 siRNA and were treated with 10 μM ORY for 96 h, ACSL4 expression (**F**) was determined by western blotting, cell viability (**G**) was detected by SRB, and the production of lipid peroxides (**H**) was analyzed by flow cytometry. **I** Quantitative ChIP studies were conducted to characterize the enrichment of H3K4me2 and H3K9me2 at promoters of *ACSL4* gene in A549 cells treated with 10 μM ORY for 96 h. *n* ≥ 3; Mean ± SEM; *P < 0.05; **P < 0.01; ***P < 0.001.

According to previous studies, ACSL4 promotes ferroptosis sensitivity by synthesizing PUFA [39], and pharmacological inhibiting of ACSL4 with thiazolidinediones has been shown to prevent cancer tissue demise in a murine model of ferroptosis [11]. Here, we examined ACSL4 expression in response to LSD1 inhibitor. All tested cell lines exhibited a significant increase of ACSL4 expression after treatment with multiple LSD1 inhibitors (Fig. 3A–C and Supplementary Fig. 4A). We also found that the mRNA level of ACSL4 was increased by ORY in a concentration-dependent manner (Fig. 3D). Consistent with this observation, the knockdown of LSD1 resulted in an upregulation of ACSL4 mRNA and protein levels (Supplementary Fig. 4B and Fig. 3E). To examine whether the increase of ACSL4 is necessary for LSD1 inhibitor-induced ferroptosis, ACSL4 was silenced with specific siRNAs (Fig. 3F). The results showed that cell viability was partially rescued by ORY when ACSL4 expression was effectively silenced (Fig. 3G and Supplementary Fig. 4C). Meanwhile, the level of lipid peroxidation induced by ORY was partially restored by knockdown of ACSL4 (Fig. 3H and Supplementary Fig. 4D). This observation suggested that ACSL4 upregulation contributes to LSD1 inhibitor-induced ferroptosis in LSD1 inhibitor-sensitive cell lines. ChIP-qPCR showed that inhibition of LSD1 increased H3K4me2 levels, but not H3K9me2 levels, at ACSL4 promoter (Fig. 3I). Taken together, our data suggested that LSD1-mediated H3K4me2 demethylation is involved in ACSL4-regulated ferroptosis.

### Decreased xCT is critical for LSD1 inhibitor-induced ferroptosis

**Fig. 4:**
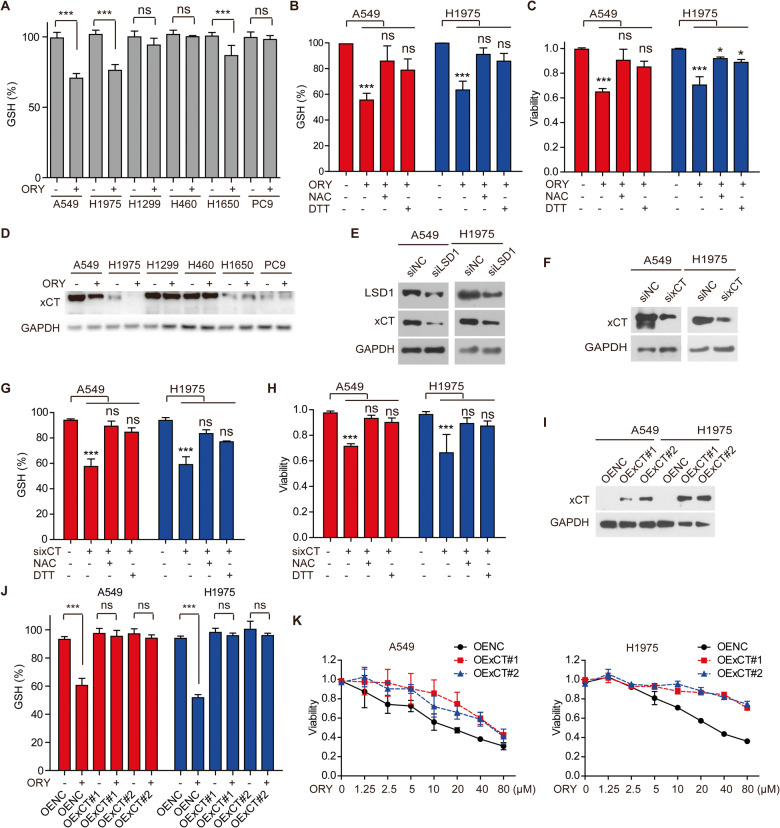
Decreased xCT is critical for LSD1 inhibitor to induce ferroptosis. **A** The levels of GSH were detected in NSCLC cell lines treated with 10 μM GSK for 96 h. **B**, **C** After A549 and H1975 cell lines were treatment with 10 μM ORY for 48 h and 5 mM NAC or 1 mM DTT was added again for 48 h as indicated, the levels of GSH (**B**) and cell viability (**C**) were measured. **D** xCT expression was determined by western blotting in NSCLC cell lines treated with 10 μM ORY for 96 h. **E** The expression of xCT and LSD1 was analyzed by western blotting in A549 and H1975 cell lines transfected with LSD1 siRNA for 48 h. **F** xCT expression was analyzed by western blotting in A549 and NCI-H1975 cell lines transfected with LSD1 siRNA for 48 h. **G**, **H** After A549 and H1975 cell lines were transfected with xCT siRNA for 48 h and 5 mM NAC or 1 mM DTT was added again for 48 h as indicated, the levels of GSH (**G**) and cell viability (**H**) were measured. **I** xCT expression was analyzed by western blotting in xCT-overexpressing cells. **J** After xCT-overexpressing cells were treated with 10 μM ORY for 48 h and 5 mM NAC or 1 mM DTT was added again for 48 h as indicated, the levels of GSH were measured. **K** Cell viability was determined by SRB in xCT-overexpressing cells treated with ORY at increasing concentrations for 96 h. *n* ≥ 3; Mean ± SEM; ^ns^*P* > 0.05; *P < 0.05; **P < 0.01; ***P < 0.001.

GSH participates in redox process by combing with phospholipid peroxides and free radicals to protect the sulfhydryl-containing proteins and enzymes from damage [7]. Therefore, a low GSH concentration is interpreted as evidence of ferroptosis [40]. In this study, we investigated GSH levels in NSCLC cell lines treated with ORY. The result showed that only the sensitive NSCLC cell lines had significantly reduced GSH content when LSD1 was inhibited (Fig. 4A and Supplementary Fig. 5A). Both NAC and DTT restored GSH and survival that had been reduced by ORY in A549 and H1975 cells (Fig. 4B, C). Given that GSH content is consistent with sensitivity of cancer cells to LSD1 inhibitor, we were inspired to speculate that GSH metabolism is the key factor in LSD1 inhibitor-induced ferroptosis in sensitive NSCLC cell lines.

The amino acid antiporter xCT plays a key role in GSH synthesis [4, 41]. Coincidentally, LSD1 inhibitor significantly reduced xCT protein and mRNA levels in A549 and H1975 cells (Fig. 4D and Supplementary Fig. 5B, C), which corresponded to a decrease in GSH levels (Fig. 4A and Supplementary Fig. 5A). In addition, knockdown of LSD1 significantly decreased xCT expression in A549 and H1975 cells (Fig. 4E). This prompted us to further examine the role of xCT in LSD1 inhibitor-induced ferroptosis. Impressively, knockdown of xCT resulted in a significant reduction in GSH and cell viability, which could be abrogated by DTT and NAC (Fig. 4F-H). Conversely, overexpression of xCT restored GSH and survival that had been reduced by ORY (Fig. 4I-K). We next investigated whether LSD1 could regulate the promoter region of *SLC7A11* gene. Our results showed that ORY did not changed the enrichment of H3K9me2 at the *SLC7A11* gene promoters (Supplementary Fig. 5D). Collectively, our data suggest that inhibition of LSD1 decreases xCT expression and xCT-dependent GSH synthesis, thereby promoting ferroptosis in sensitive NSCLC cell lines.

### LSD1 inhibitor decreases xCT by inhibiting ATF4 in sensitive NSCLC cell lines

**Fig. 5:**
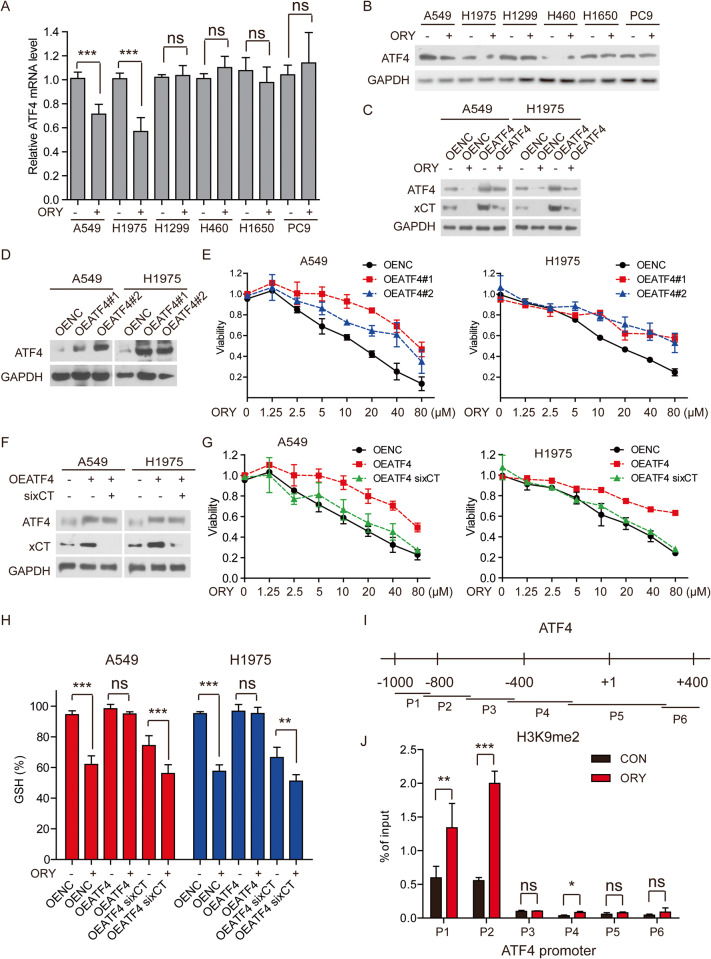
LSD1 inhibition reduces xCT expression via ATF4. **A** ATF4 mRNA levels were determined by qRT-PCR in NSCLC cell lines treated with 10 μM ORY for 96 h. **B** ATF4 expression was determined by western blotting in NSCLC cell lines treated with 10 μM ORY for 96 h. **C** The expression of xCT and ATF4 were measured by western blotting in ATF4-overexpressing A549 and H1975 cells treated with 10 μM ORY for 96 h. **D** ATF4 expression were measured by Western blotting in ATF4-overexpressing A549 and H1975 cells. **E** Cell viability was determined by SRB assay in ATF4-overexpressing A549 and H1975 cells treated with ORY at increasing concentrations for 96 h. **F** The expression of xCT and ATF4 were measured by western blotting in ATF4-overexpressing A549 and H1975 cells transfected with xCT siRNA. **G**, **H** After ATF4-overexpressing A549 and H1975 cells were transfected with xCT siRNA and were treated with ORY, cell viability (**G**) and the levels of GSH (**H**) were detected. **I** ChIP primers were designed spanning from −1000 to +400 bp around the transcription start sites of ATF4. **J** Quantitative ChIP studies were conducted to characterize the enrichment of H3K4me2 and H3K9me2 at promoters of *ATF4* gene in A549 cells treated with 10 μM ORY for 96 h. *n* ≥ 3; Mean ± SEM; ^ns^*P* > 0.05; *P < 0.05; **P < 0.01; ***P < 0.001.

To further investigate how ORY leads to xCT downregulation, we measured the regulators of xCT such as ATF4 [42], activating transcription factor 3 (ATF3) [43], aryl hydrocarbon receptor nuclear translocator-like (ARNTL) [44], NRF2 [45], BTB domain and CNC homolog 1 (BACH1) [46], STAT1 [47], STAT3 [48] and P53 [49]. The results showed that LSD1 inhibitor dramatically decreased the protein and mRNA level of ATF4 only in A549 and H1975 cells (Fig. 5A, B), which is consistent with the result that ORY affected xCT expression in sensitive NSCLC cell lines (Fig. 4D and Supplementary Fig. 5B, C). In addition, ORY did not affect the mRNA or protein levels of ATF3, ARNTL, BACH1, NRF2, and P53 (Supplementary Fig. 6A, B). We also demonstrate that inhibition of LSD1 reduced p-STAT3, which contrasts with earlier studies that showed pharmacologically suppressing STAT3 activation significantly increased xCT mRNA and protein levels [48]. To ascertain the relationship between xCT and ATF4, we transfected A549 and H1975 cells with plasmids expressing xCT and ATF4, respectively. xCT was significantly increased in ATF4-overexpressing cell lines (Fig. 5C), whereas ATF4 protein levels were unchanged in xCT-overexpressing cell lines (Supplementary Fig. 6C). In addition, overexpression of ATF4 restored xCT expression downregulated by ORY, but overexpression of xCT did not restore ATF4 expression downregulated by ORY (Fig. 5C and Supplementary Fig. 6C). Therefore, xCT is downstream of ATF4 and is regulated by ATF4 in sensitive NSCLC cells.

We further investigated the role of ATF4 in LSD1 inhibitor-induced ferroptosis. The result showed that overexpression of ATF4 reversed ORY-induced cell death (Fig. 5D, E). However, this reversal was abolished when xCT was knocked down in ATF4-overexpressing cells (Fig. 5F, G), indicating that xCT acts as an ATF4 executor to exert ferroptosis inhibition. In addition, GSH reduction by ORY was reversed by ATF4 overexpression, but was declined when xCT was knocked down in ATF4-overexpressing cells (Fig. 5H). Overall, these results further support the idea that inhibition of LSD1 can effectively decrease xCT expression by inhibiting ATF4, thereby reducing GSH synthesis and ultimately causing ferroptosis.

To further investigate how LSD1 regulates ATF4, we investigated the level of H3K4me2 and H3K9me2 at promoter of *ATF4* gene. LSD1-mediated demethylation of H3K9me2 is known to be associated with transcriptional activation of target genes [19], whereas LSD1 demethylates H3K4me2 leading to the repression of target genes [50]. The results showed that inhibition of LSD1 increased H3K9me2 levels, but not H3K4me2 levels, at ATF4 promoter (Fig. 5I, J and Supplementary Fig. 6D). In conclusion, these data indicated that LSD1 inhibitor repressed ATF4 transcription via H3K9me2 modification.

### LSD1 inhibitor acts synergistically with RSL to enhance ferroptosis

**Fig. 6:**
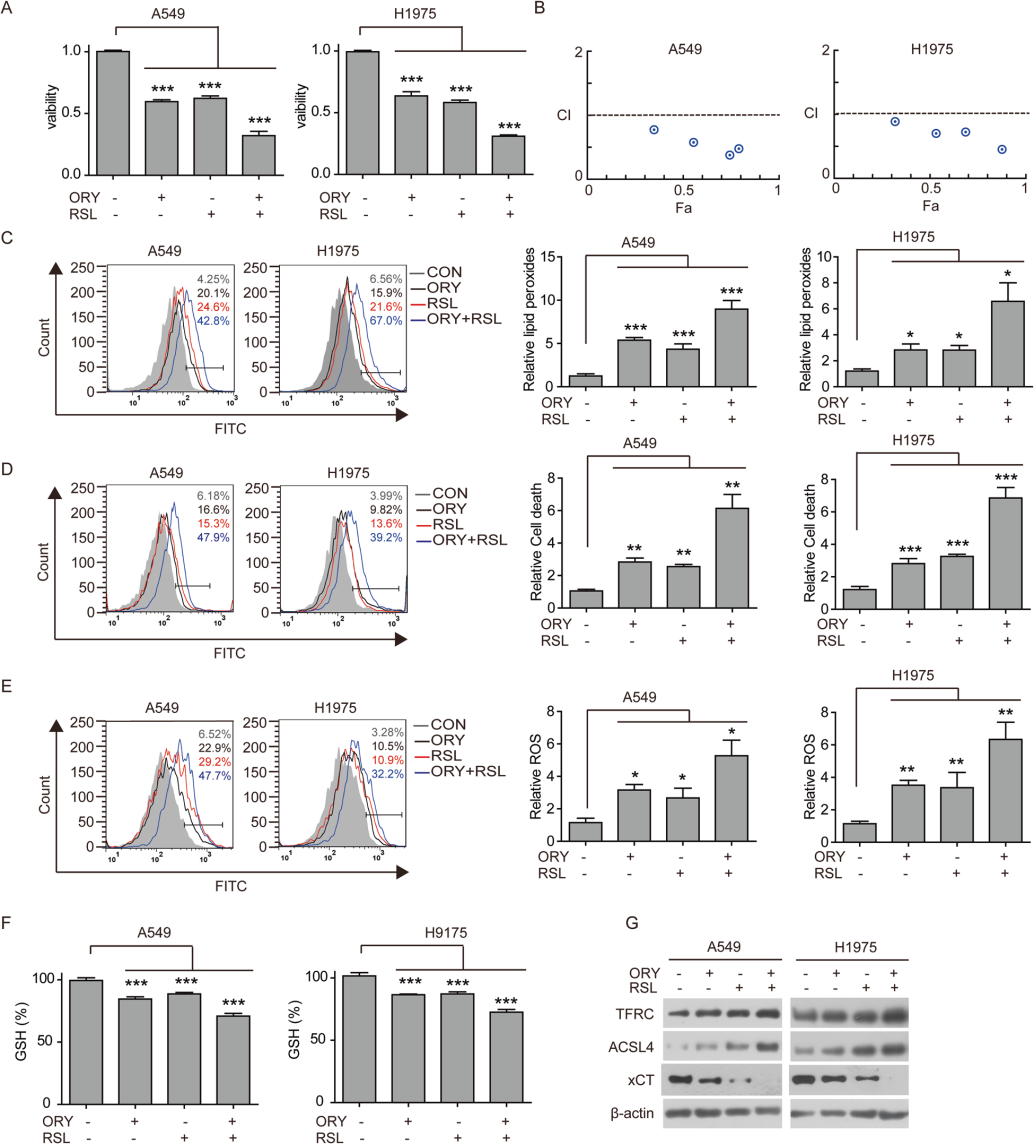
LSD1 inhibition sensitizes cancer cells to ferroptosis. **A** Cell viability was determined by SRB in A549 and H1975 cells treated with 10 μM ORY for 96 h and 2 μM RSL for 48 h alone or in combination as indicated. **B** The synergistic effect of ORY combination with RSL was analyzed in A549 and H1975 cell lines. Combination index (CI) values were calculated at the drug concentration of ORY (2.5, 5, 10, 20 μM) plus RSL (1, 2, 4, 8 μM) using the Chou-Talalay method. **C**–**G** After A549 and H1975 cells were treated with 10 μM ORY for 96 h and 2 μM RSL for 48 h alone or in combination as indicated, production of lipid peroxides (**C**), cell death (**D**), and total ROS accumulation (**E**) were analyzed by flow cytometry, the levels of GSH (**F**) were detected by Total Glutathione Assay Kit, and the expression of TFRC, ACSL4 xCT, and ATF4 (**G**) was analyzed by western blotting. *n* ≥ 3; mean ± SEM; *P < 0.05; **P < 0.01; ***P < 0.001.

Previous studies have shown that the PARP inhibitor olaparib enhances RSL-induced ferroptosis by suppressing xCT-mediated GSH biosynthesis and significantly sensitized cancer cells to RSL in a synergistic manner [51]. We next sought to determine whether LSD1 inhibition can sensitize cancer cells to ferroptosis induced by RSL. The results showed that the combination of ORY and RSL significantly suppressed cell viability in comparison to ORY or RSL alone (Fig. 6A and Supplementary Fig. 7A). The Chou-Talalay CI equation was used to calculate the CI value [52]. The curve indicated that the combination of ORY and RSL had a synergistic inhibitory effect on cell growth, with the CI values ranging below 1.0 (Fig. 6B). Thus, these results suggest that the inhibition of LSD1 sensitizes cancer cells to RSL-induced ferroptosis.

We further investigate the effect of ORY and RSL on ferroptosis. The results showed that the combination of ORY and RSL significantly increased lipid peroxidation and ROS accumulation and significantly decreased cell viability compared to single treatment (Fig. 6C–E and Supplementary Fig. 7B–D). We also found the combined effects of ORY and RSL in reducing GSH (Fig. 6F and Supplementary Fig. 7E). These results indicate that combining ORY and RSL can promote ferroptosis. Interestingly, RSL increased the expression of ACSL4 and TFRC, which was further upregulated when combined with ORY (Fig. 6G and Supplementary Fig. 7F). And again, compared to ORY or RSL treatment alone, ORY plus RSL more significantly inhibited xCT expression (Fig. 6G and Supplementary Fig. 7F). Taken together, these data demonstrate that the inhibition of LSD1 significantly enhances RSL-mediated ferroptosis.

### Combination of LSD1 inhibitor and RSL exerts synergistic anti-tumor growth in vivo

**Fig. 7:**
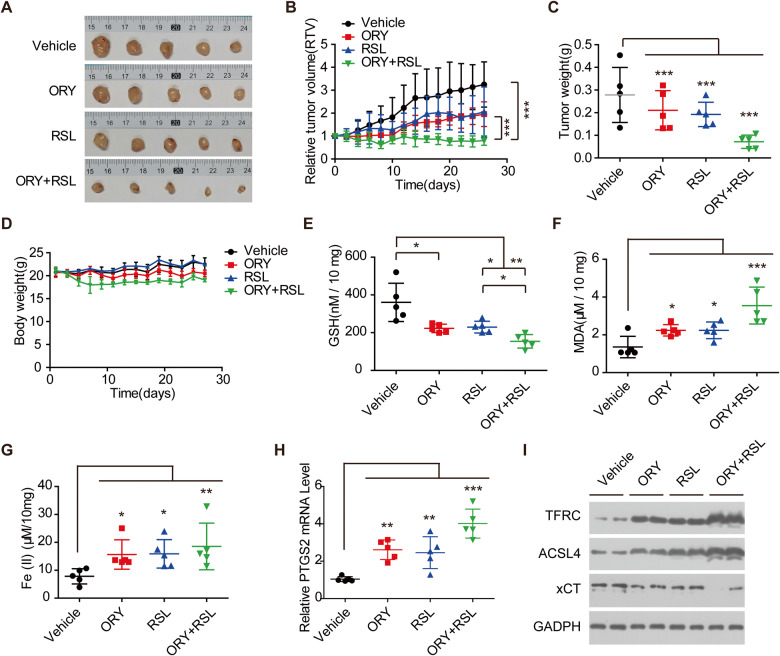
LSD1 inhibitors can enhance the tumor suppressive effect of ferroptosis inducers in vivo. **A** Images of xenograft tumors. **B** Relative tumor volume during the administration period. **C** Tumor weight. **D** Body weight of mice. **E** The content of GSH in tumor tissue. **F** The content of MDA in tumor tissue. **G** The content of Fe^2+^ in tumor tissue. **H** The mRNA levels of PTGS2 in tumor tissue. **I** Western blotting analysis of TFRC, ACSL4, and xCT in tumor tissue. *n* ≥ 3; mean ± SEM; *P < 0.05; **P < 0.01; ***P < 0.001.

Next, we examined the in vivo efficacy of ORY and RSL using nude mice xenograft models. We observed that ORY and RSL impaired the growth of xenograft tumors, respectively, which was consistent with that reported previously [53]. Importantly, the combination of ORY and RSL resulted in synergistic benefits (Fig. 7A–C). ORY and RSL treatment did not cause any significant weight loss in our animal studies, suggesting that the treatment was well tolerated in vivo (Fig. 7D).

To investigate the mechanism of synergy between ORY and RSL, we extracted tumor tissue to examine the factors involved in ferroptosis. The result showed that ORY and RSL resulted in attenuation of GSH, overproduction of MDA, Fe^2+^, and PTGS2 mRNA, respectively (Fig. 7E-H). Coincidentally, ORY plus RSL resulted in even less GSH, more MDA, Fe^2+^, and PTGS2 than either agent alone (Fig. 7E–H). Further analyses revealed that treatment with RSL or ORY alone only had a moderate effect on ACSL4 and TFRC levels in tumors, but their combination resulted in a potent rise of ACSL4 and TFRC levels (Fig. 7I). Additionally, ORY can synergize with RSL to suppress the expression of xCT in established xenograft tumors. These data collectively suggested that inhibiting LSD1 acts synergistically with RSL to enhance ferroptosis in vivo.

## Discussion

Studies have shown that LSD1 generates detectable ROS in response to DNA damage [54], while in other studies LSD1 has been shown to inhibit ROS production when levels are excessive or inappropriate [55]. This phenomenon may help to explain the inconsistent effects of LSD1 on various tumor treatments [56]. In this study, we have shown that inhibition of LSD1 can lead to the accumulation of intracellular ROS and lipid hydroperoxides in tumor cells, ultimately resulting in ferroptosis. Notably, while the reduction of xCT expression is the key factor for LSD1 inhibitor-induced ferroptosis, upregulated ACSL4 expression and increased intracellular iron levels are also required. We have also highlighted enhanced anti-tumor effects by combination of LSD1 inhibitor and RSL in vitro and in vivo.

xCT plays a critical role in glutamine metabolism and regulates ferroptosis in cancer cells [5]. In this study, we found that LSD1 inhibitors affect GSH metabolic processes within tumor cells by inhibiting xCT transcription, thereby revealing a new mechanism for LSD1 regulating ferroptosis. Previous studies have shown that xCT is regulated by various proteins such as P53 [49], ARNTL[44], NRF2 [57], ATF3 [43], STAT3 [48], and BACH1 [46]. However, our findings indicate that ORY does not alter the expression of these regulators. While previous studies have shown that ATF4 can increase xCT levels [42], our findings confirmed that ORY increases the transcription-repressive mark H3K9me2 at the ATF4 promoter, ultimately repressing the transcription of ATF4, reducing xCT expression, and inducing ferroptosis.

Previous studies have indicated that iron is important in ferroptosis [58], and that altered iron homeostasis may be an important factor in the pathogenesis of cancer [59]. The iron load may be affected by the altered expression of certain iron metabolism-associated proteins [60]. As shown here, inhibition of LSD1 upregulated TFRC protein levels and subsequent intracellular Fe^2+^ amounts. Indeed, TFRC is a prognostic biomarker associated with tumor burden and survival in lung adenocarcinoma patients [61]. Compared with normal tissues, the expression of TFRC is remarkably downregulated in tumor tissues [61]. Although we demonstrated that knockdown of TFRC inhibited ferroptosis induced by LSD1 inhibitors, TFRC was even increased in cells without obvious ferroptosis, indicating that other cells may have other mechanisms for ferroptosis tolerance. The transcription factor special protein 1 (SP1) was an essential factor that could bind to the TFRC promoter and upregulate the TFRC transcription [62]. However, our results indicate that TFRC expression is independent of SP1 activity (Supplementary Fig. 3F). In addition, we found that inhibition of LSD1 reduced YAP expression (Supplementary Fig. 3G), which was different from earlier studies that YAP promotes TFRC transcription [30], demonstrating that ORY-regulated TFRC is independent of YAP in the cell line we have examined. Our findings confirmed that ORY increases H3K4me2 at the TFRC promoter, ultimately promoting the transcription of TFRC and inducing ferroptosis.

As a pivotal indicator and regulator of ferroptosis, ACSL4 modulates the cellular lipid composition [63]. In the present study, we found that LSD1 suppression increased ACSL4 expression. The previous study has shown that the expression of ACSL4 appears to be a predictive marker for ferroptosis sensitivity in different cellular contexts [39]. Notably, ACSL4 suppression reversed the effect of LSD1 inhibitor on cell death and lipid peroxidation in A549 and H1975 cells, implying that ferroptosis induced by LSD1 inhibitors was mediated through ACSL4. However, we found that ORY induced ACSL4 elevation in both LSD1 inhibitor-sensitive and -insensitive cell lines, indicating that ACSL4 is not a predictive marker for ferroptosis induced by LSD1 inhibitors in NSCLC. Recent studies indicate that SP1 and YAP were crucial transcription factors that promoted ACSL4 transcription by binding to the *ACSL4* promoter region [30, 64]. However, LSD1 regulates ACSL4 protein levels independent of SP1 and YAP in the cell line we have examined (Supplementary Fig. 3F, G). Our research findings have confirmed that ORY enhances H3K4me2 at the ACSL4 promoter, consequently stimulating the transcription of ACSL4 and triggering ferroptosis.

**Fig. 8:**
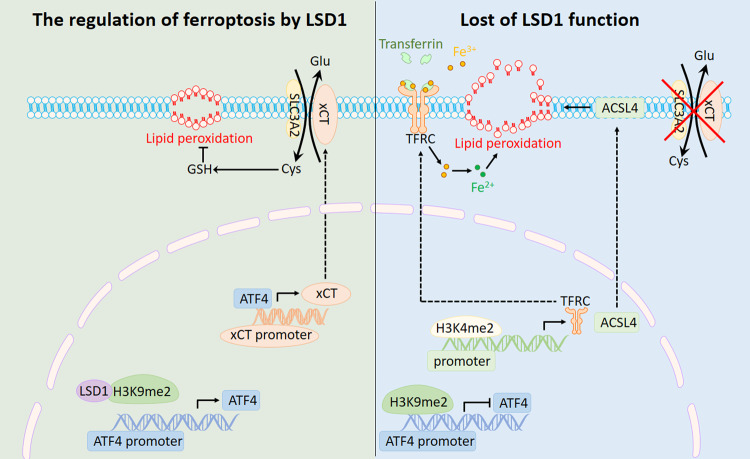
A working model. Proposed working model of inhibiting LSD1 on ferroptosis in cancer cells. (Left) LSD1 enhances the demethylation of H3K9me2 at the ATF4 promoter and activates ATF4 expression, which leads to the upregulation of xCT, thereby inhibiting ferroptosis in cancer cells. (Right) Inhibition of LSD1 results in the gain of H3K9me2 at ATF4 promoters, which silences *ATF4* gene expression, leading to the reduction of xCT and ferroptosis. In addition, the suppression of LSD1 leads to an increase in H3K4me2 levels at the promoter sequences of TFRC and ACSL4, which promotes the expression of TFRC and ACSL4, which will promote the synthesis of unsaturated fatty acid and the accumulation of intracellular iron, respectively, thus promoting ferroptosis.

In conclusion, we reveal new mechanisms of LSD1 regulating ferroptosis and highlight the synergistic anti-tumor effects of ferroptosis inducer in combination with LSD1 inhibitors. Our findings suggest that pharmacologically blocking LSD1 can promote ferroptosis by increasing H3K9me2 at the *ATF4* promoter, leading to inhibition of ATF4 transcription and xCT expression. Additionally, inhibition of LSD1 can activate transcription of TFRC and ACSL4, which promotes the accumulation of intracellular ferrous irons and synthesis of unsaturated fatty acid leading to ferroptosis. The mechanisms by which LSD1 inhibition regulating ferroptosis are summarized as Fig. 8. Our results may contribute, at least in part, to the understanding of how cancer cells protect themselves from oxidative stress through LSD1.

### Supplementary information

Supplementary Materials

Original Data File

Reproducibility checklist

## Data Availability

All data are available within the article and supplementary files, or from the authors upon reasonable request.
