# Supplementary material for: Inhibition of LSD1 induces ferroptosis through the ATF4-xCT pathway and shows enhanced anti-tumor effects with ferroptosis inducers in NSCLC
Source: Cell Death Dis. 2023 Nov 3;14(11):716. doi: 10.1038/s41419-023-06238-5 (PMC10624898; doi:10.1038/s41419-023-06238-5)

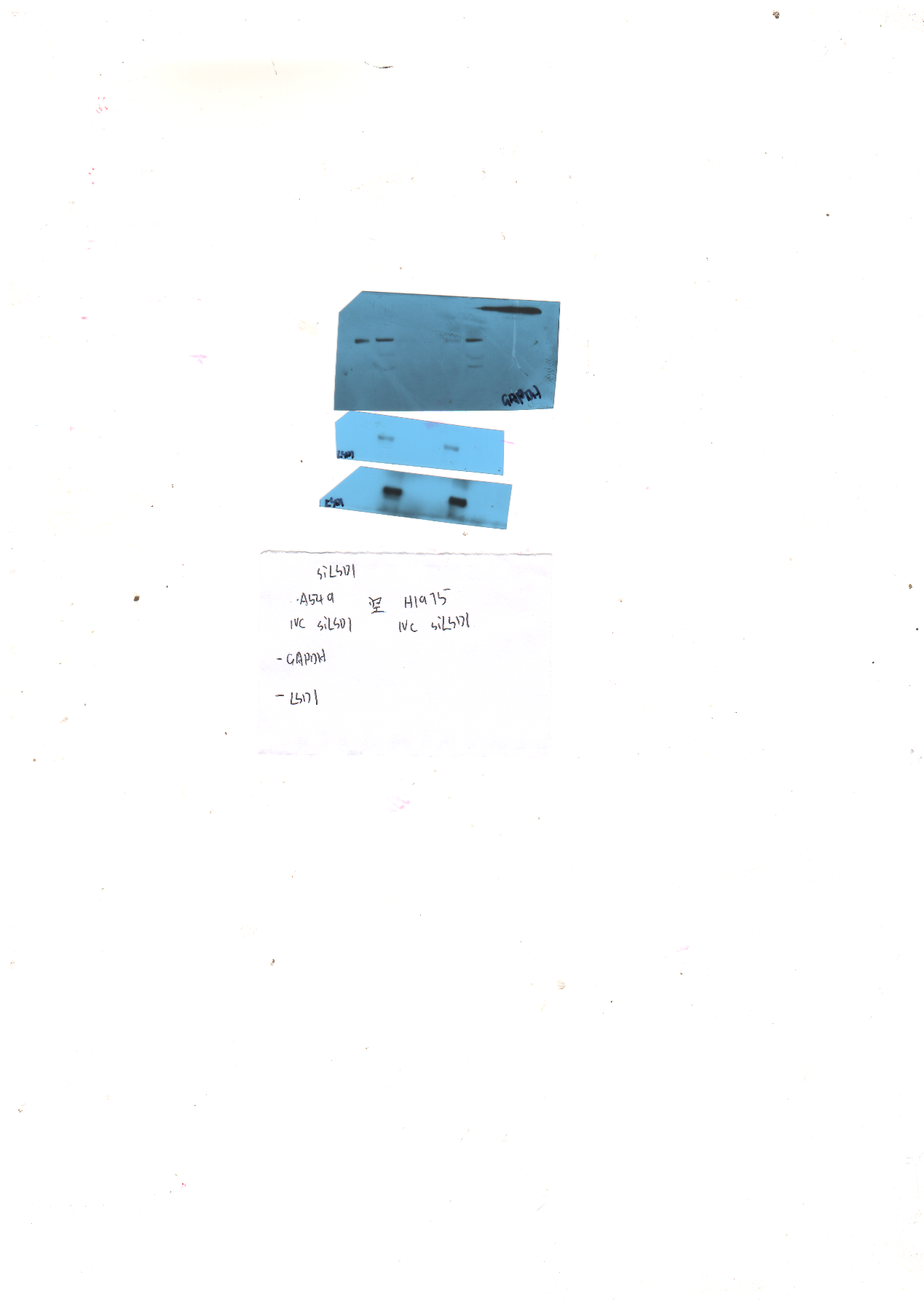
Fig. 1C


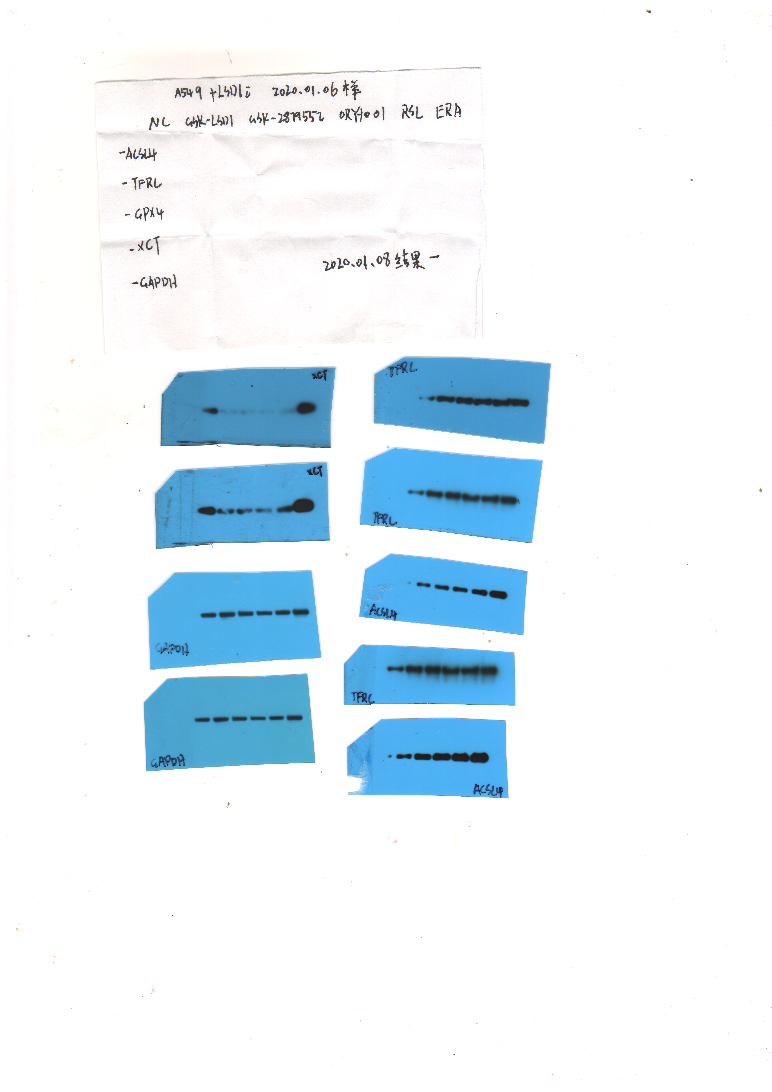

Fig. 2E


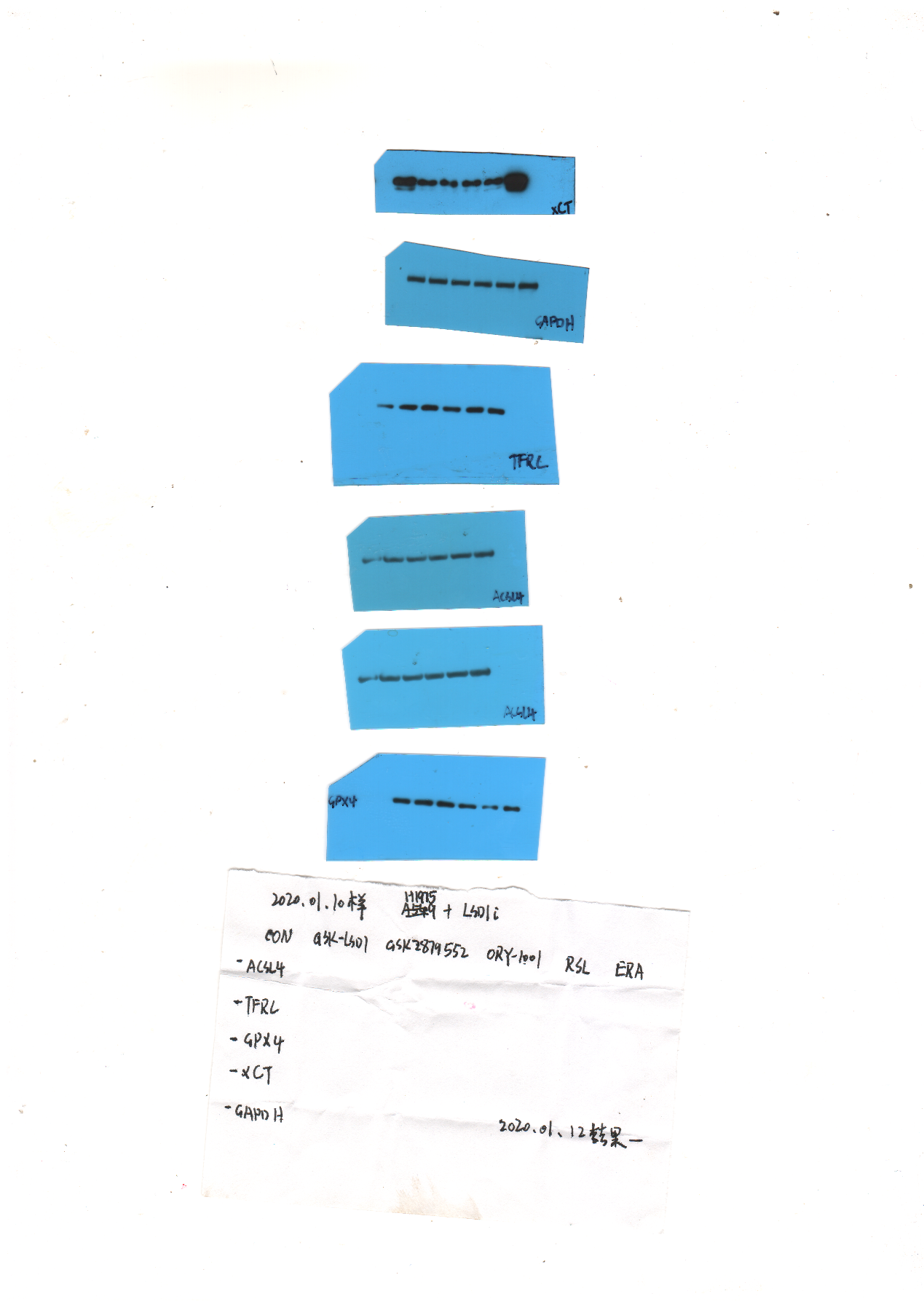


Fig. 2F


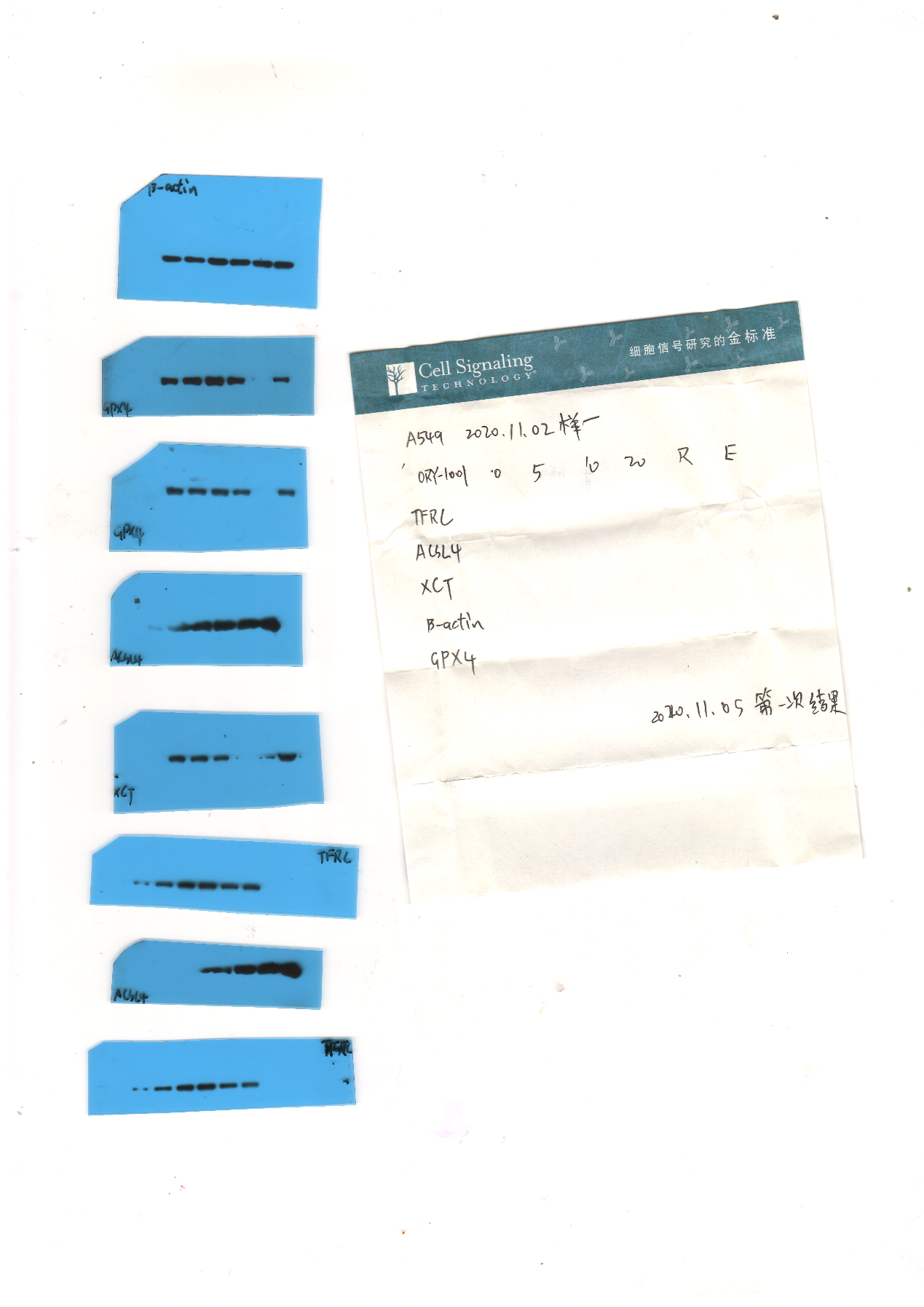


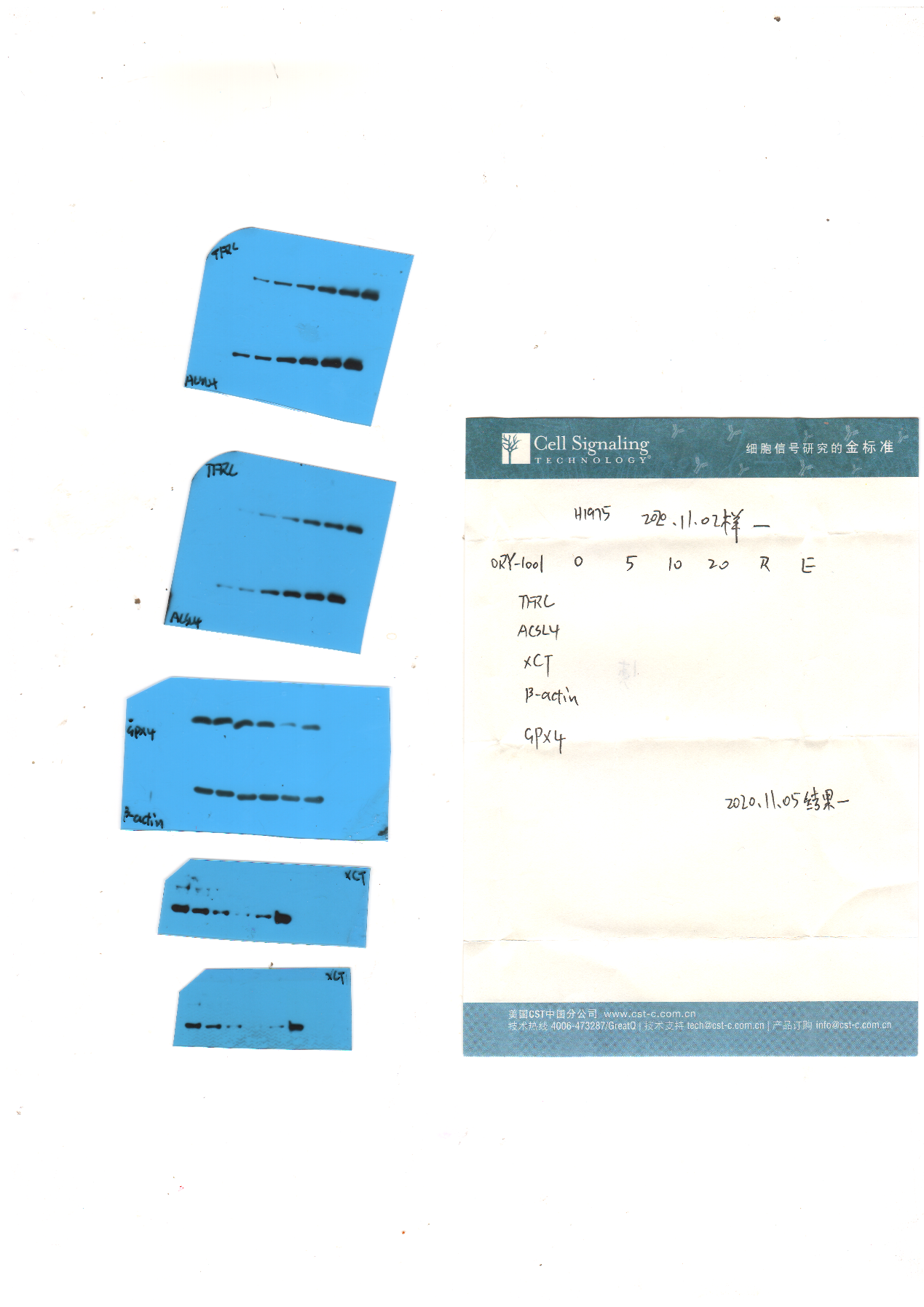


Fig. 3A


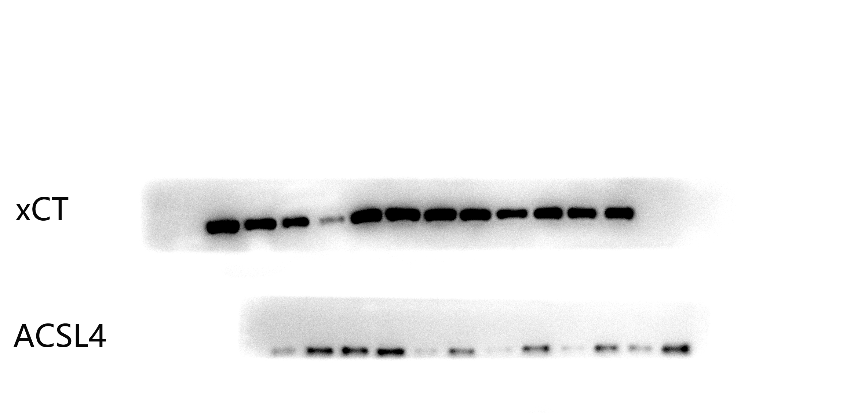


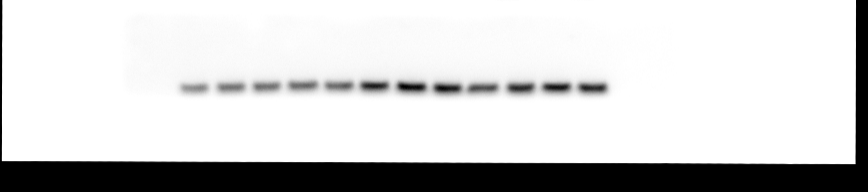


Fig. 3B


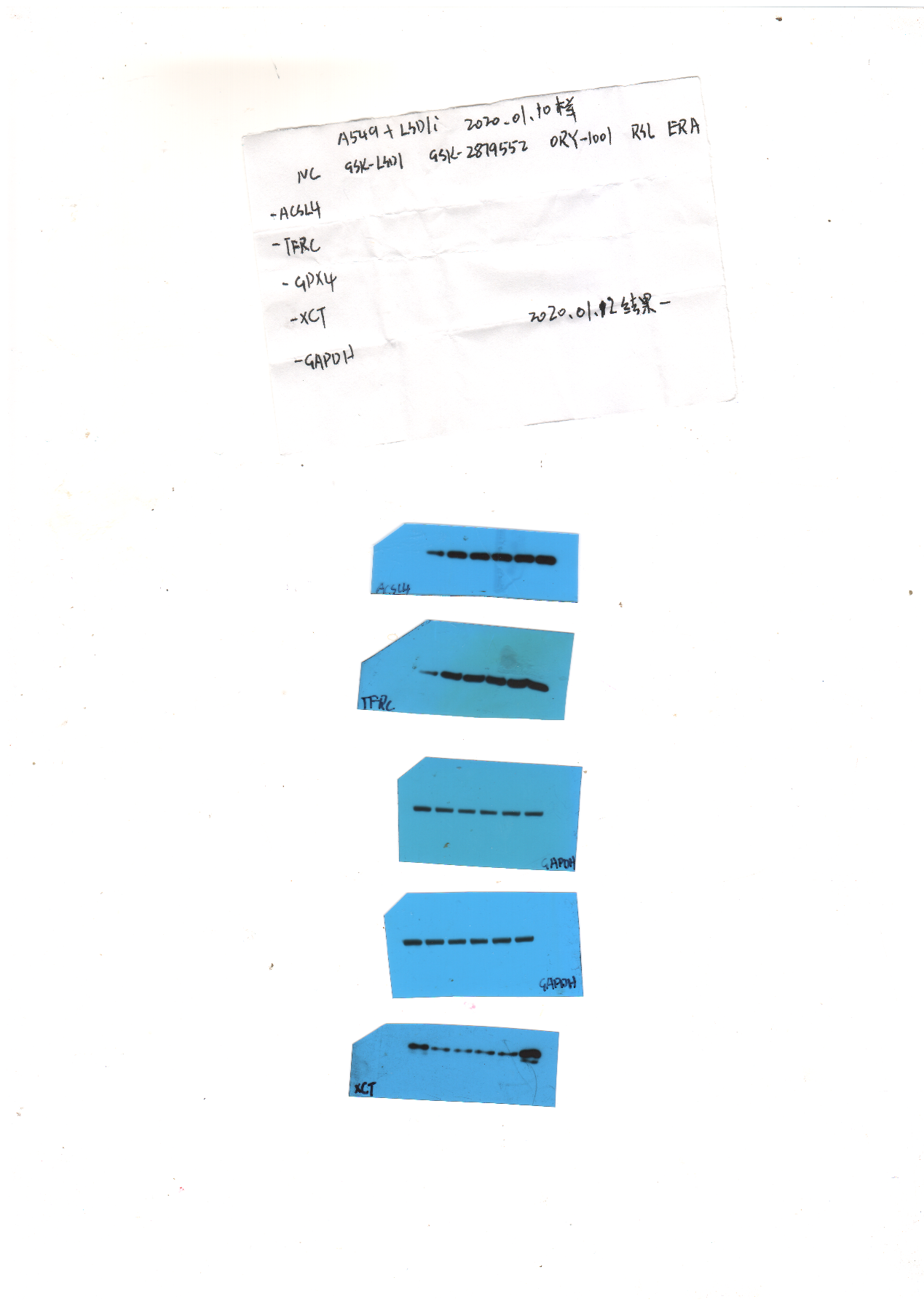


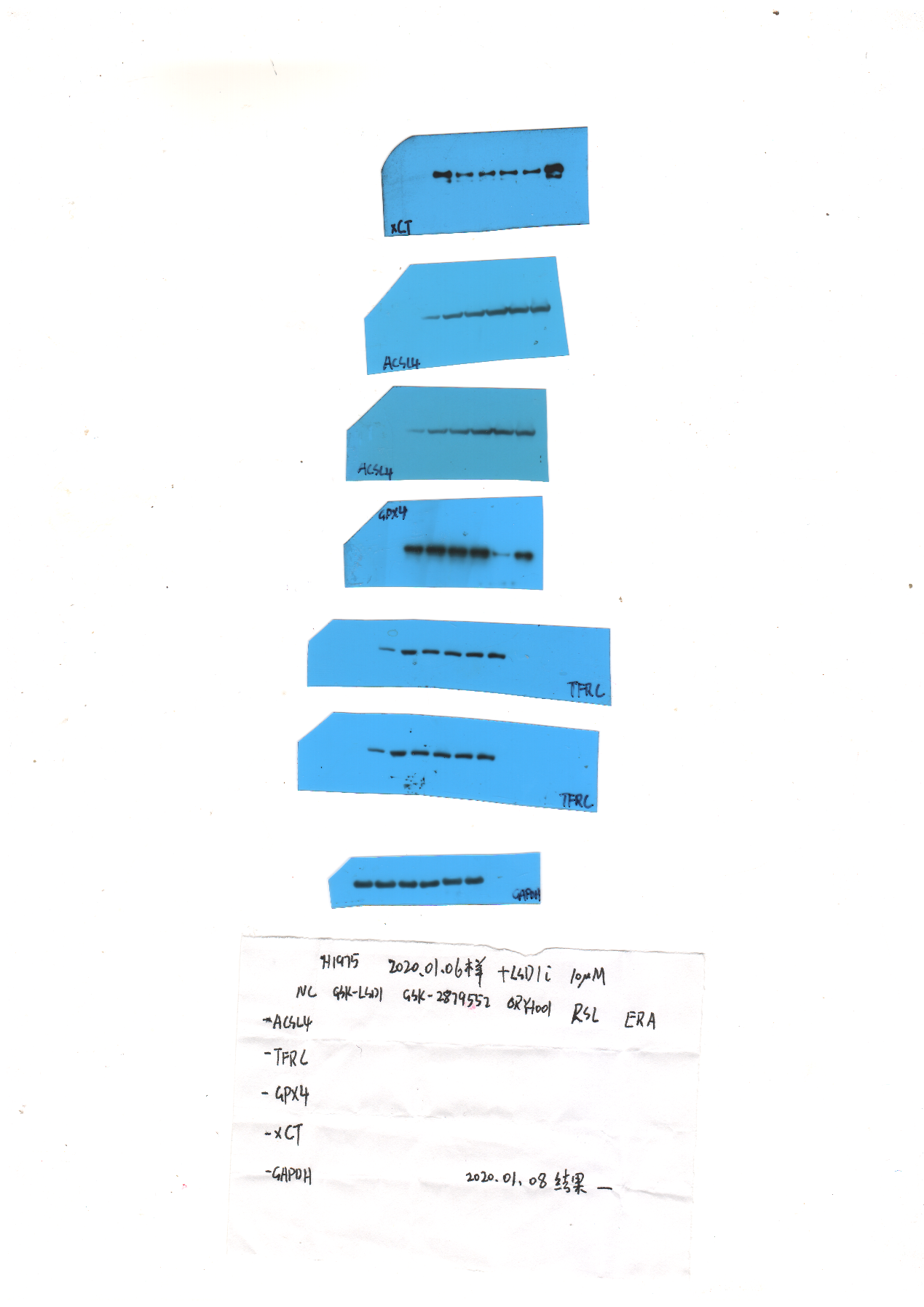


Fig. 3C


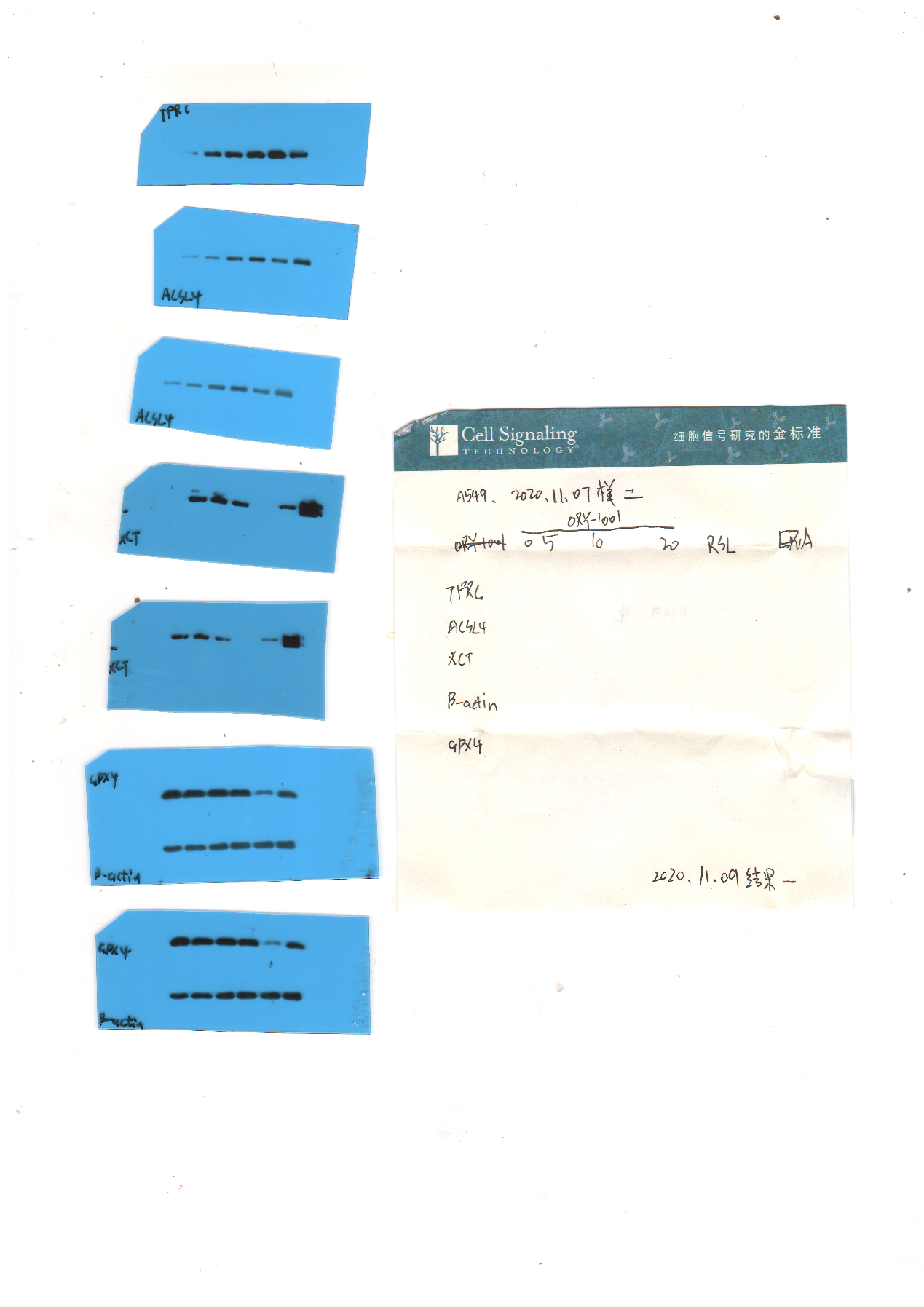


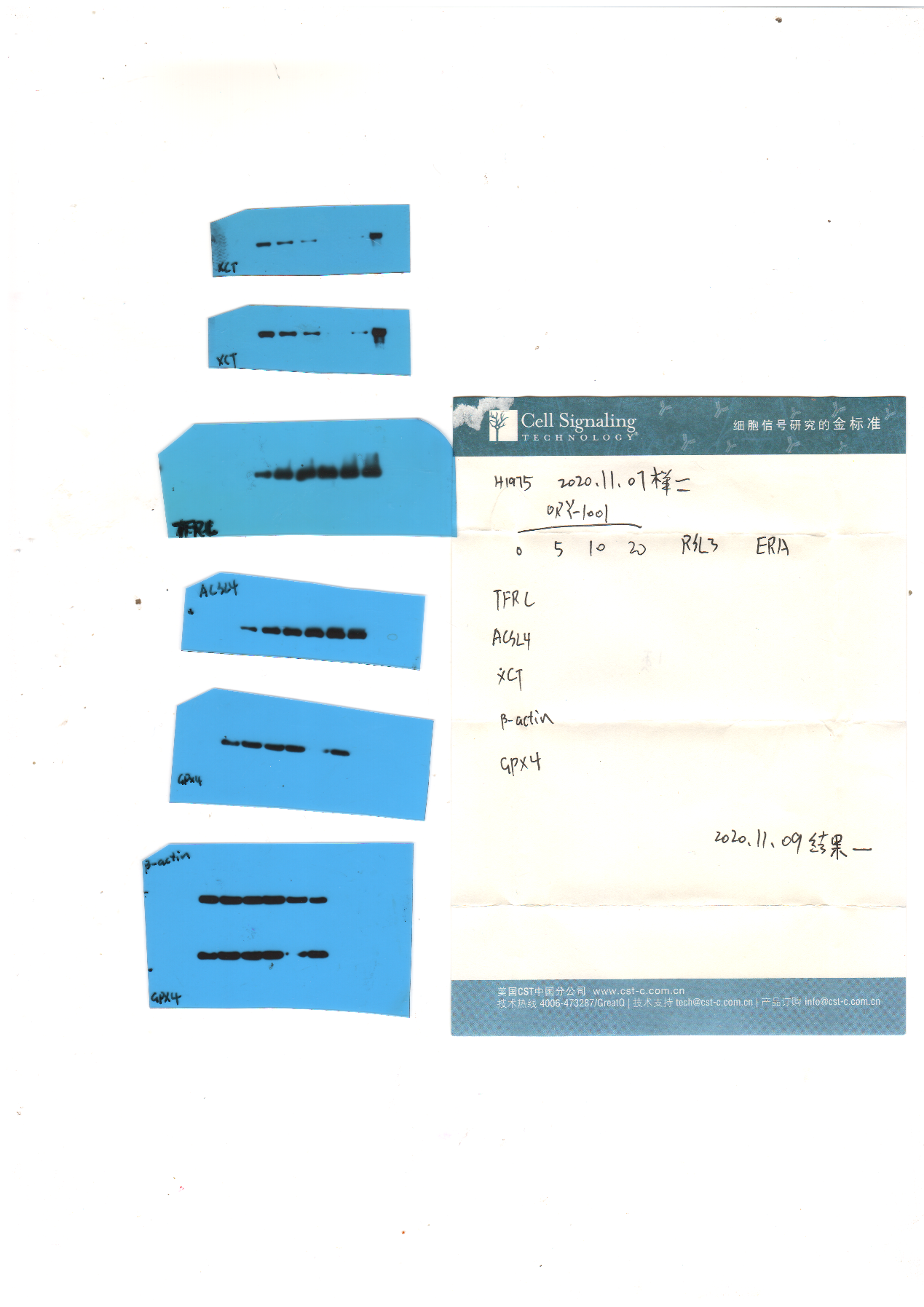


Fig. 4D


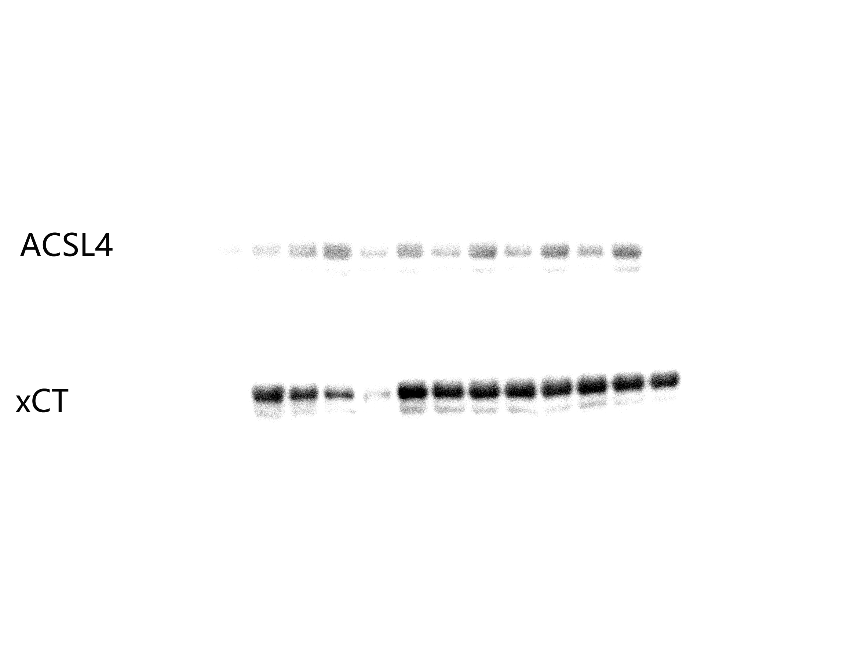


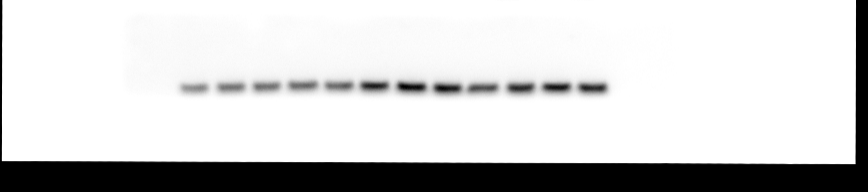


Fig. 4F


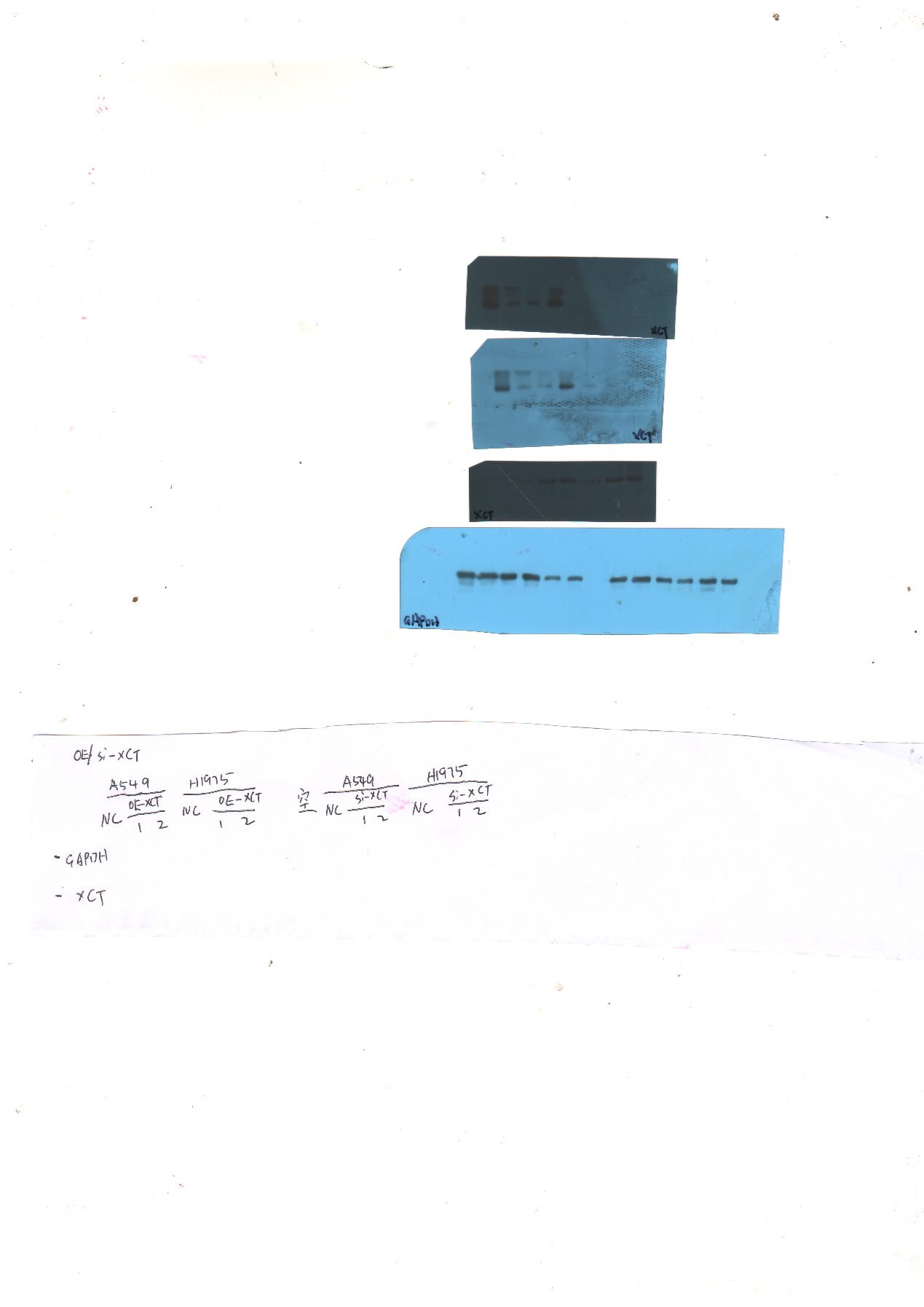


Fig. 4I


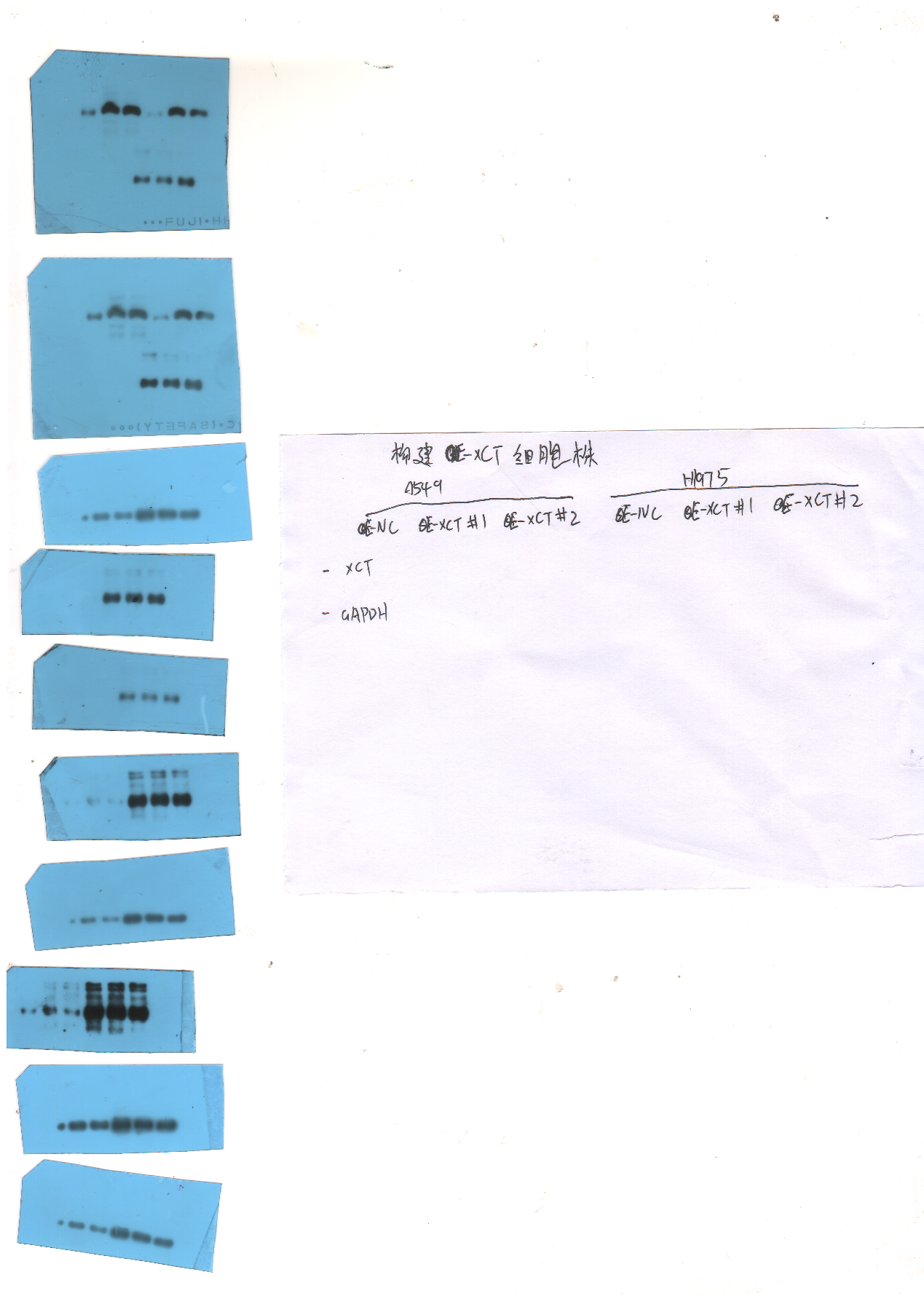


Fig. 5B


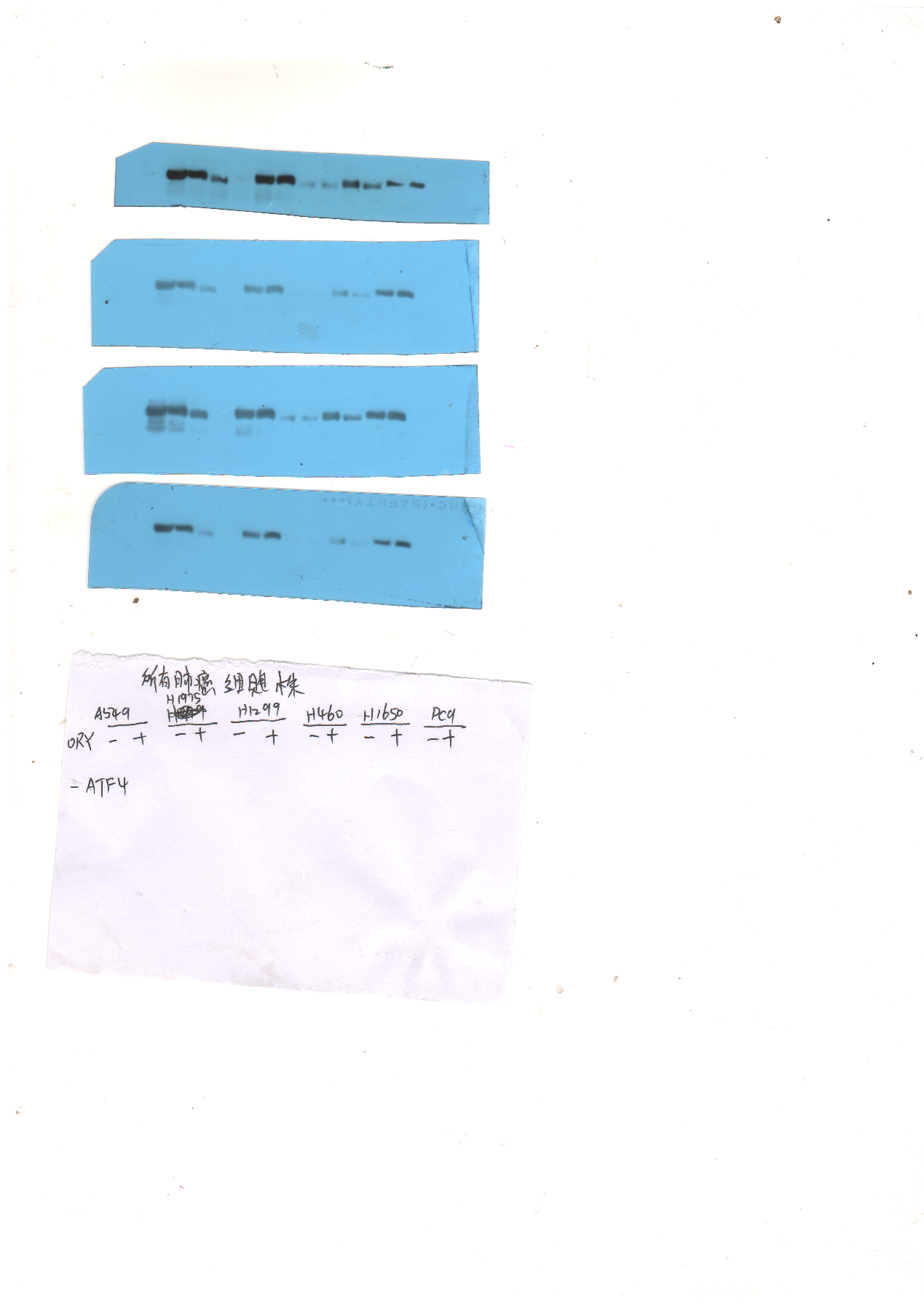


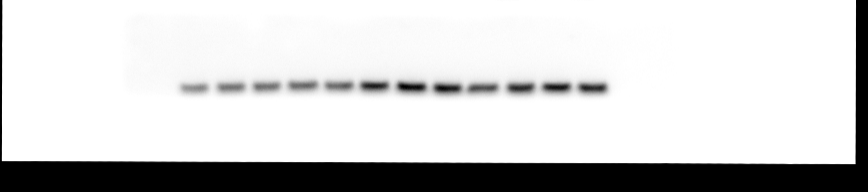


Fig. 5C


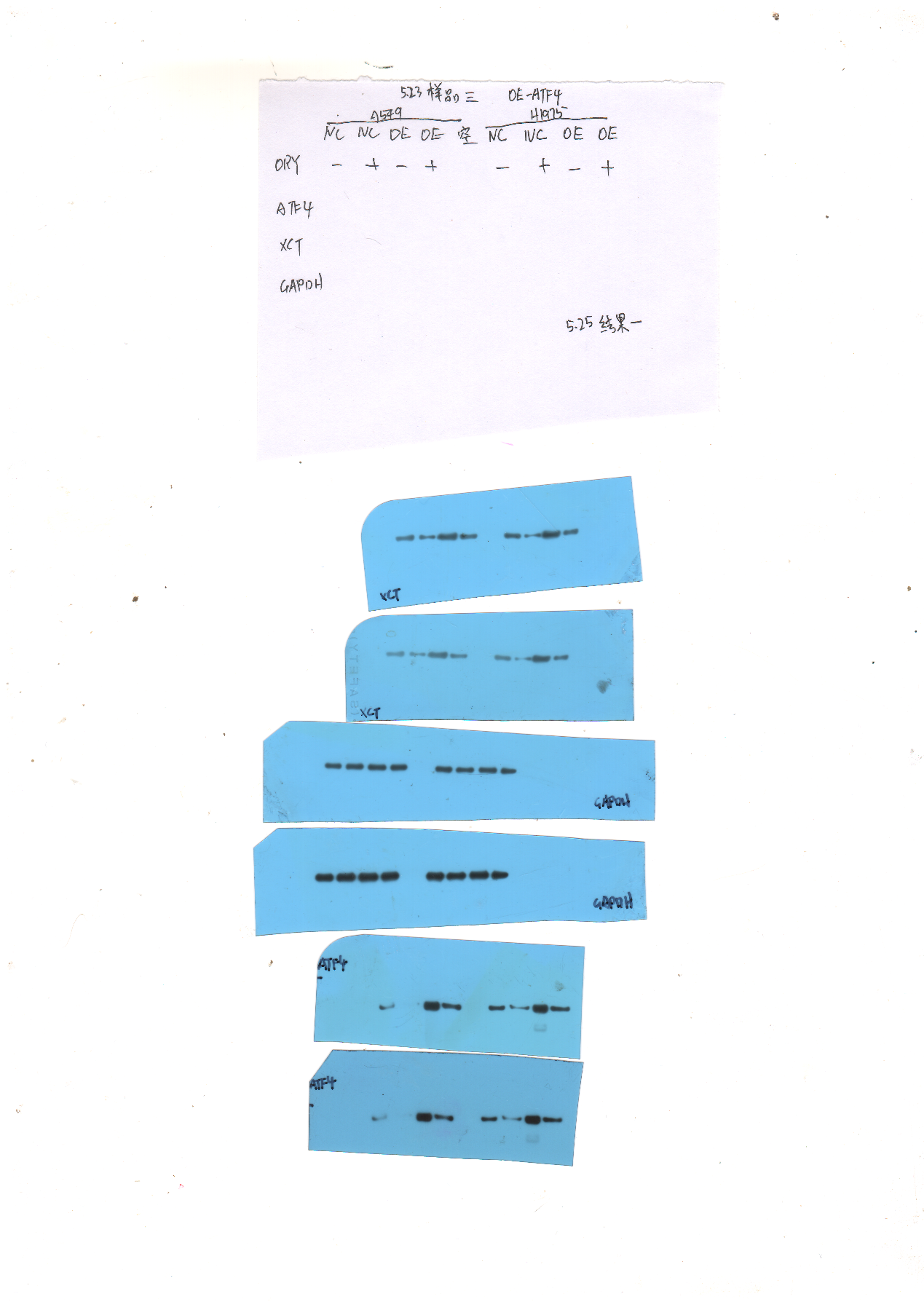


Fig. 5D


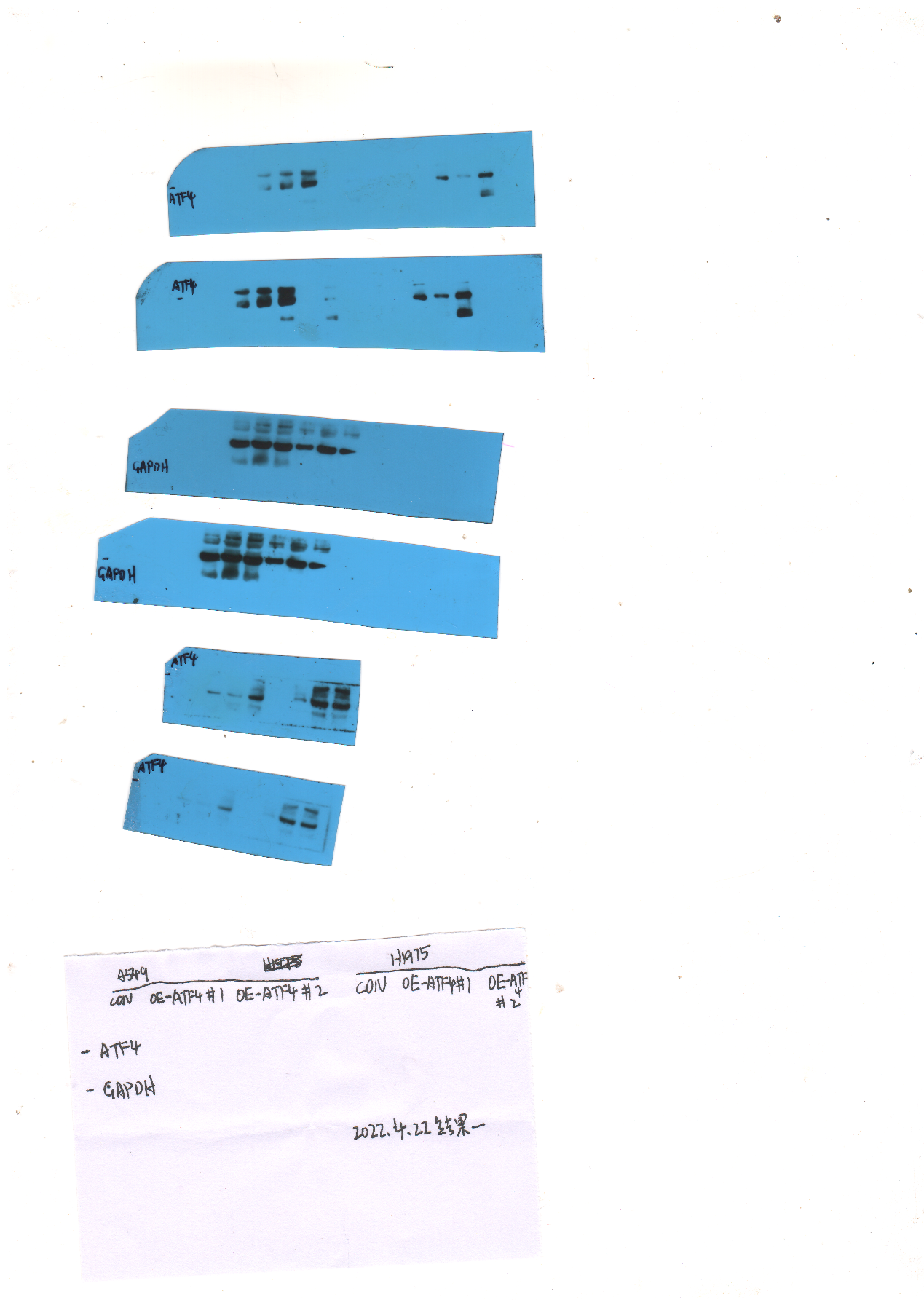


Fig. 5F


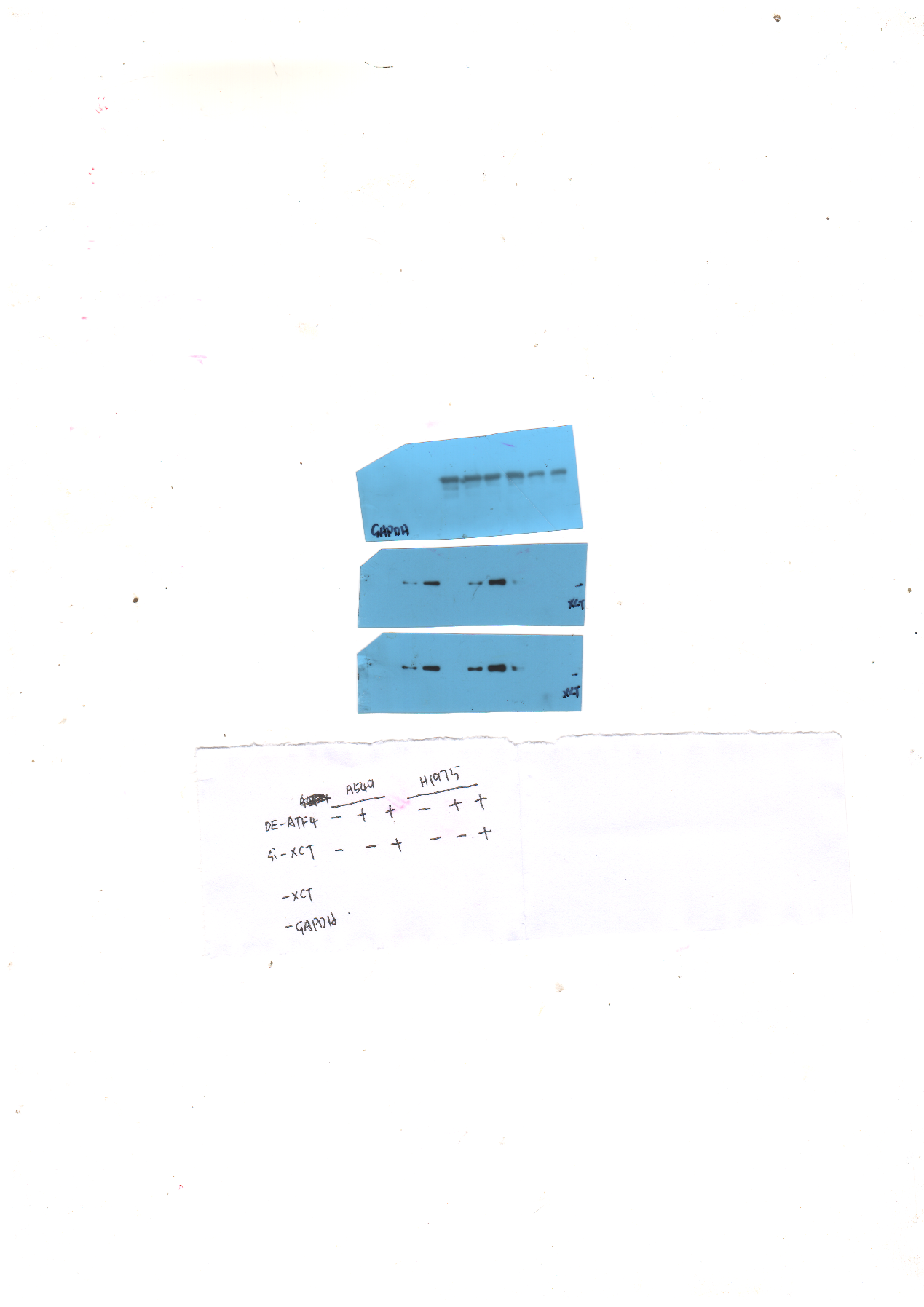


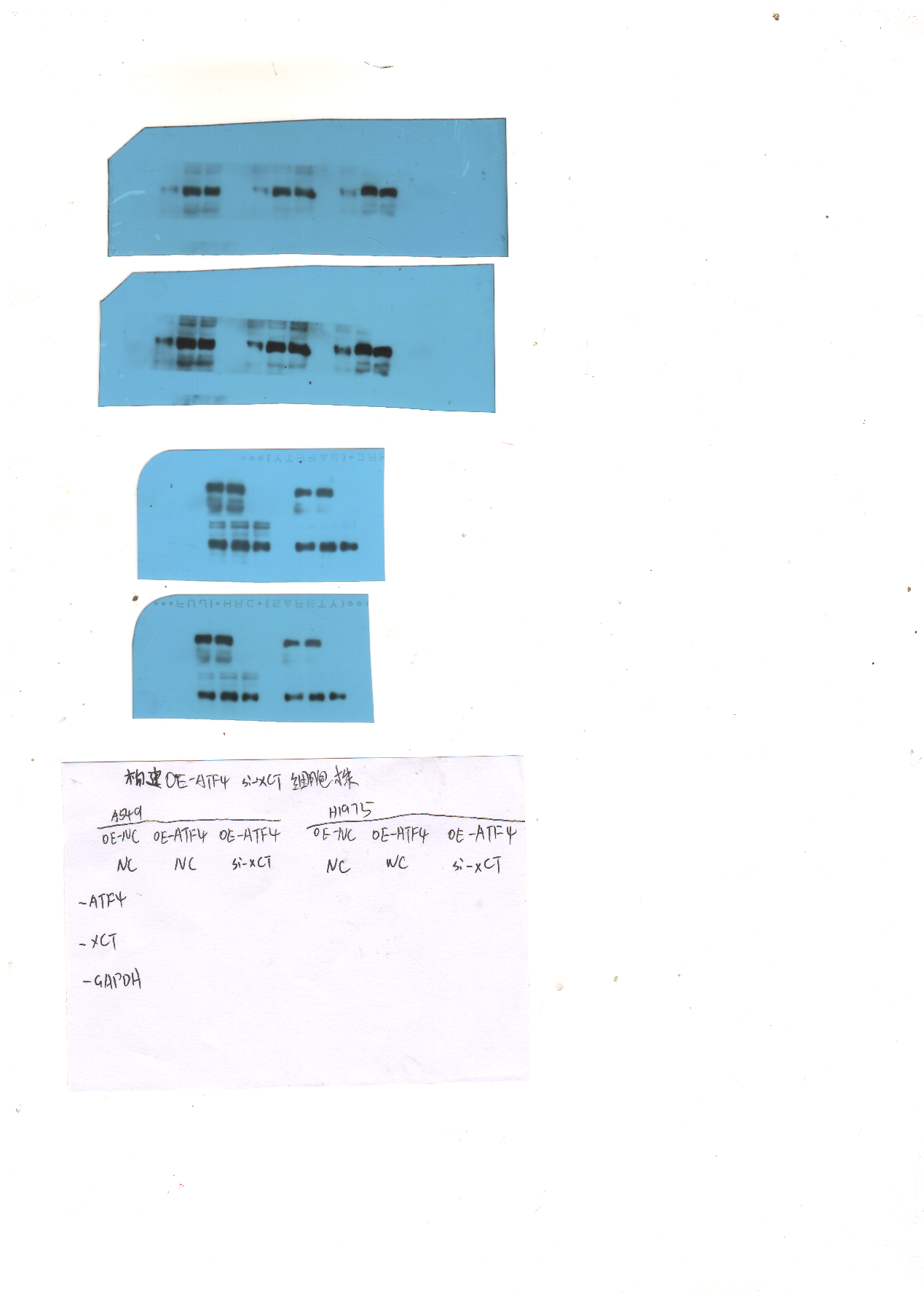


Fig. 6G


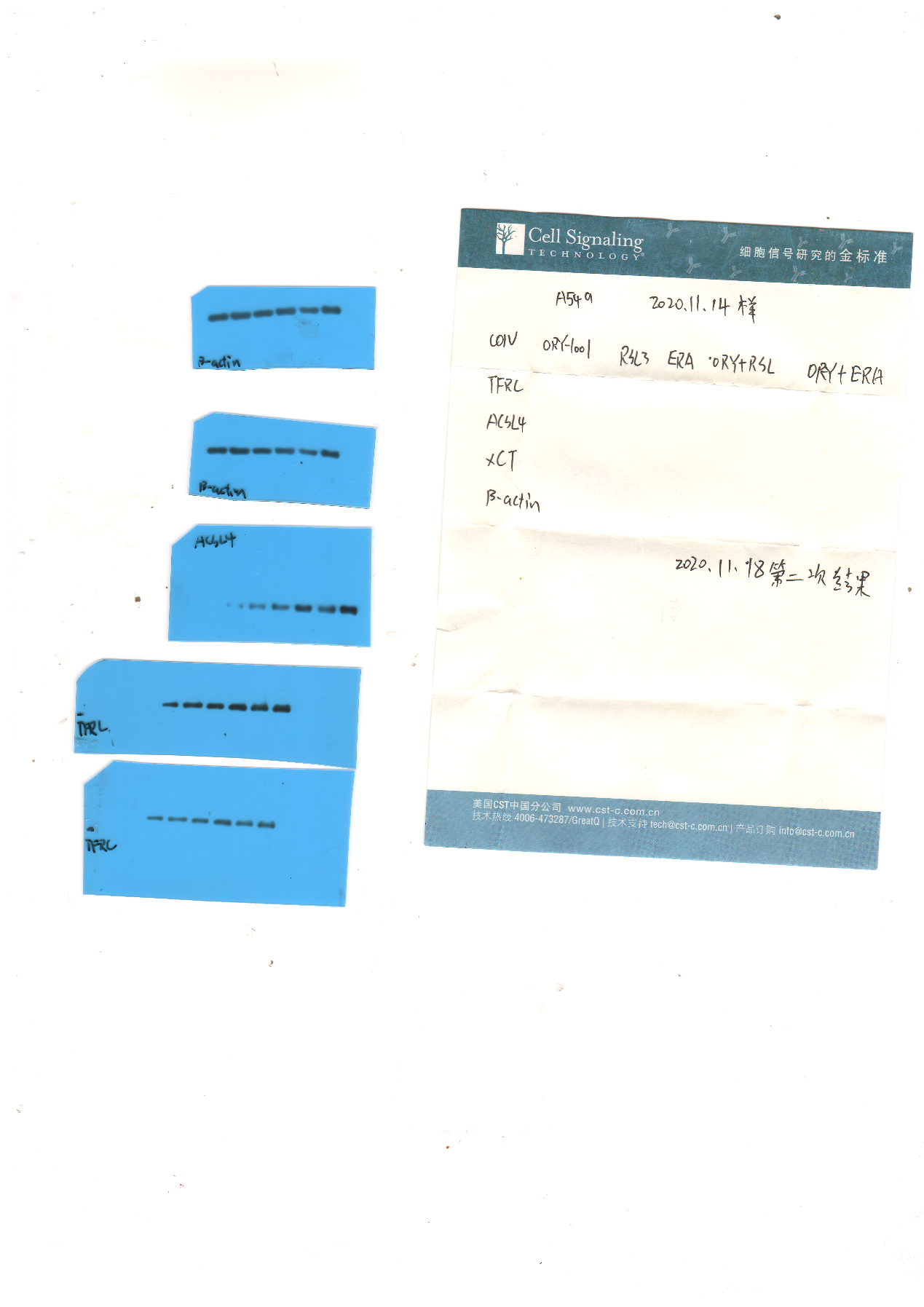


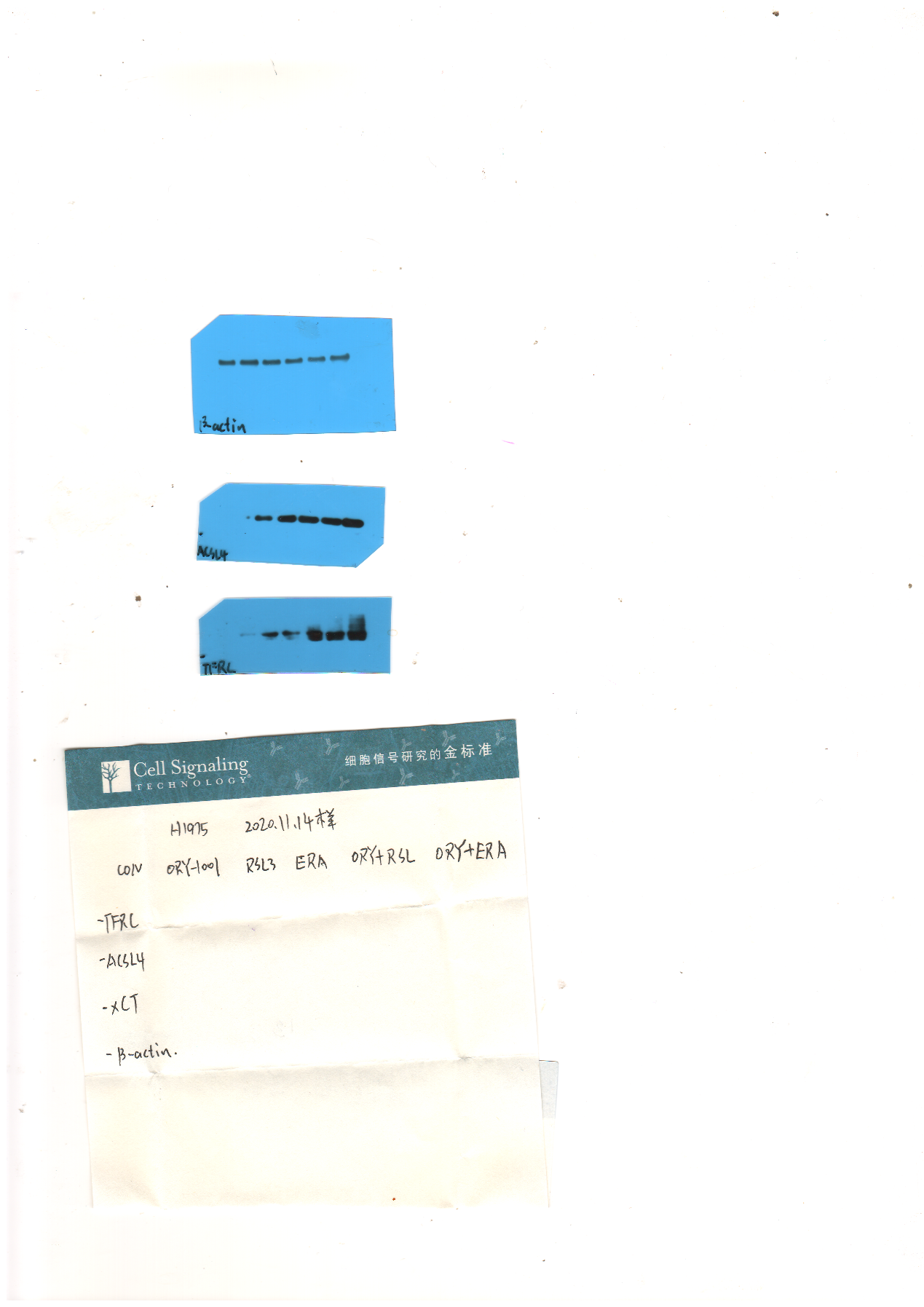


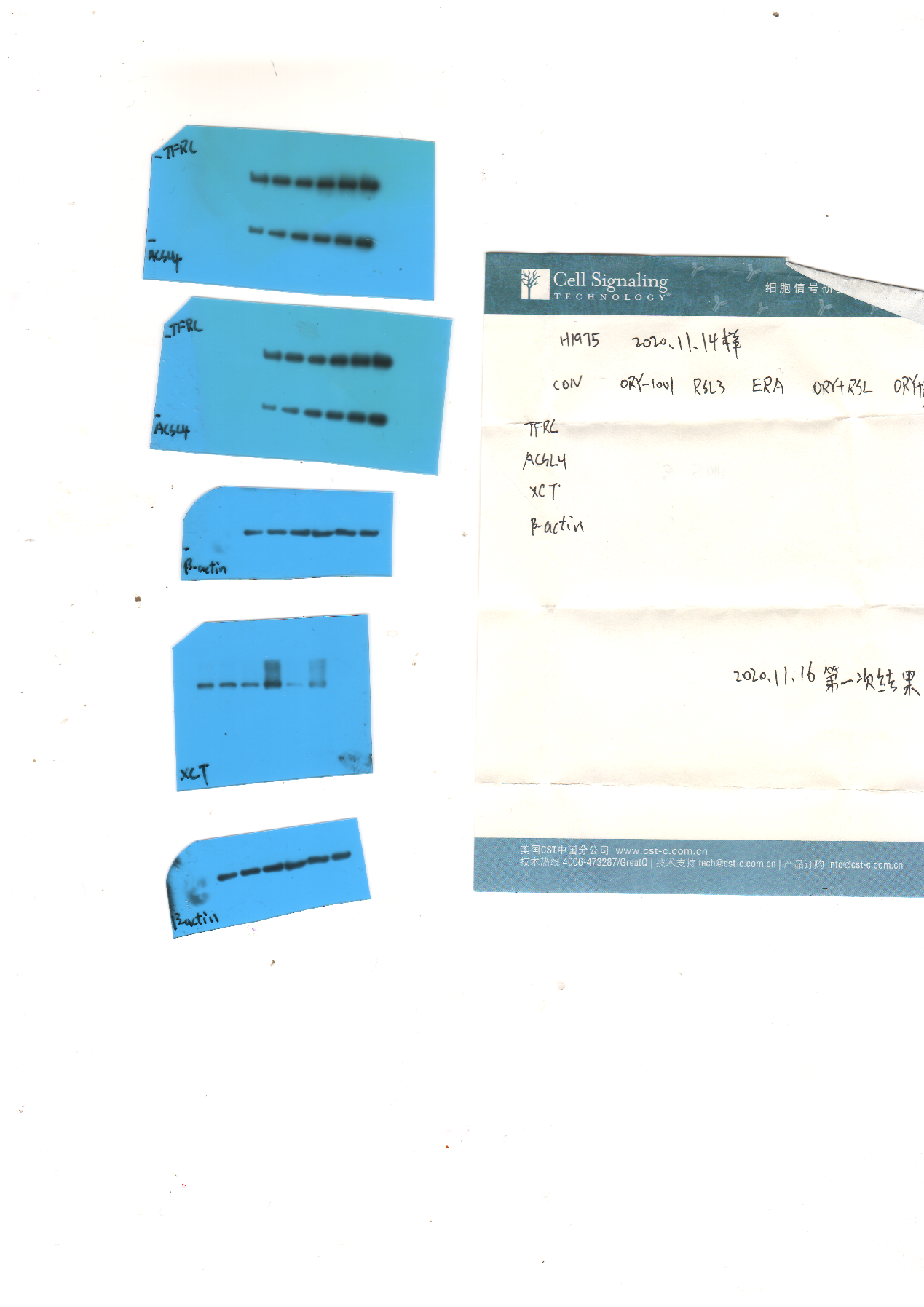


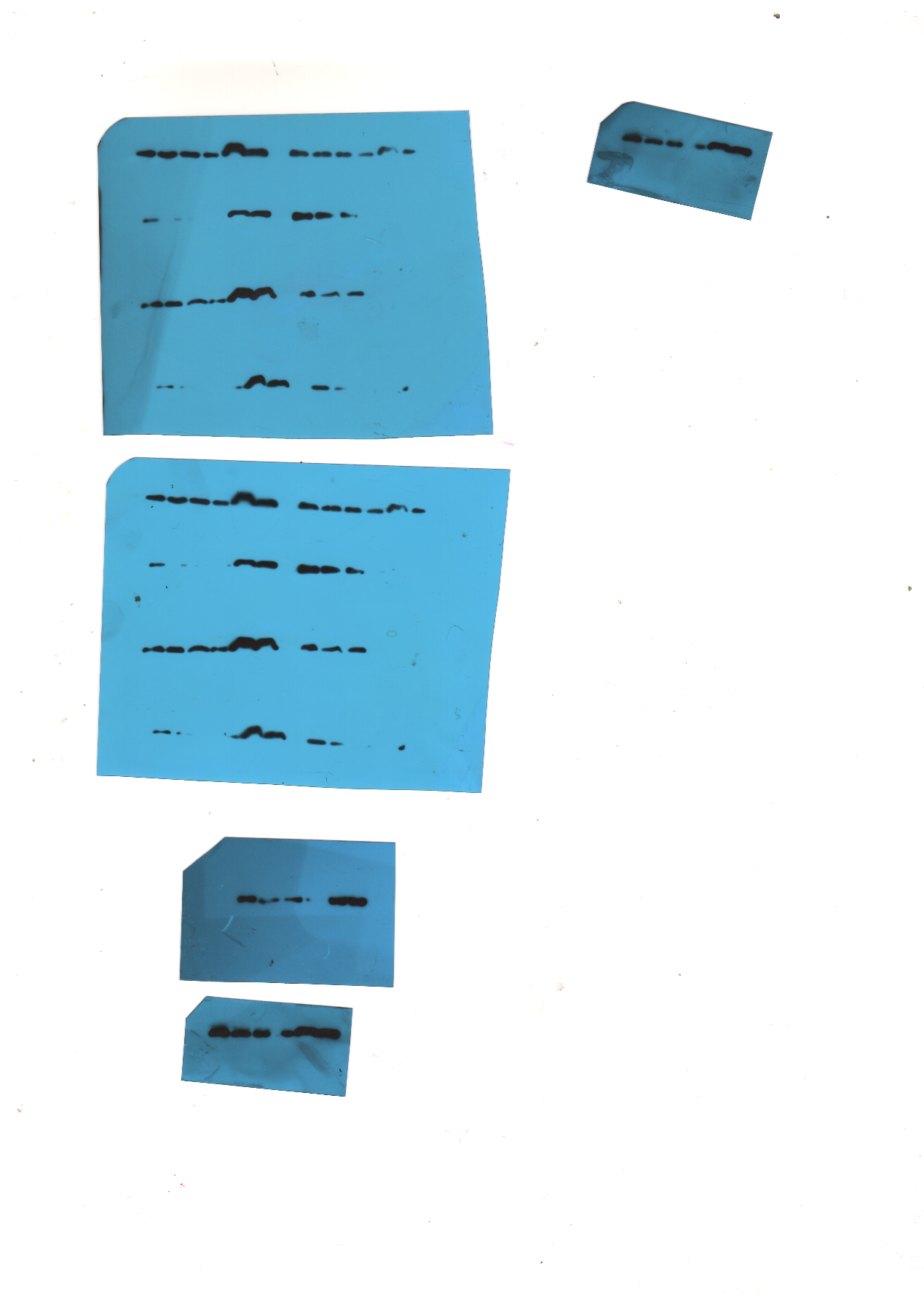


Fig. 7F


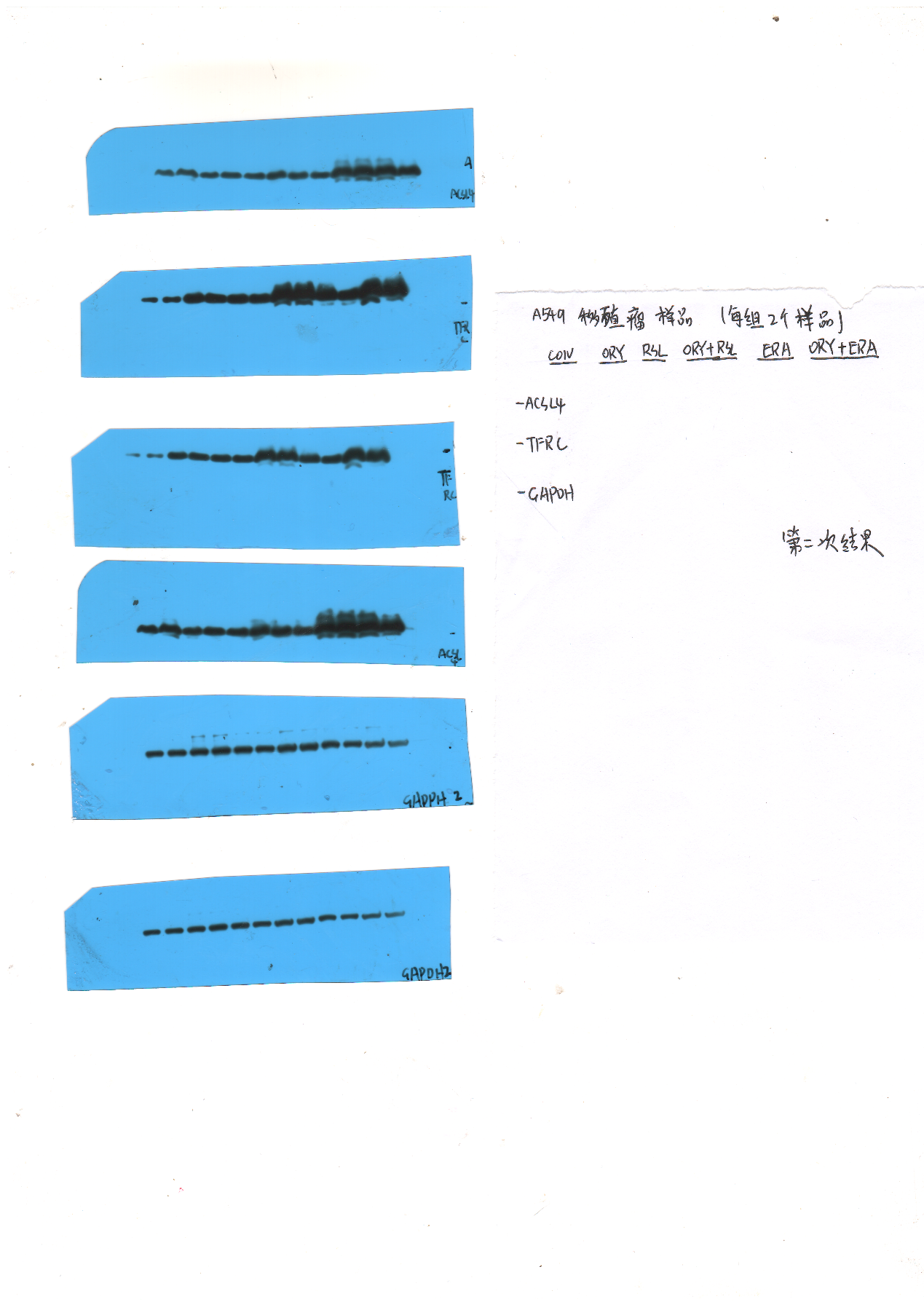


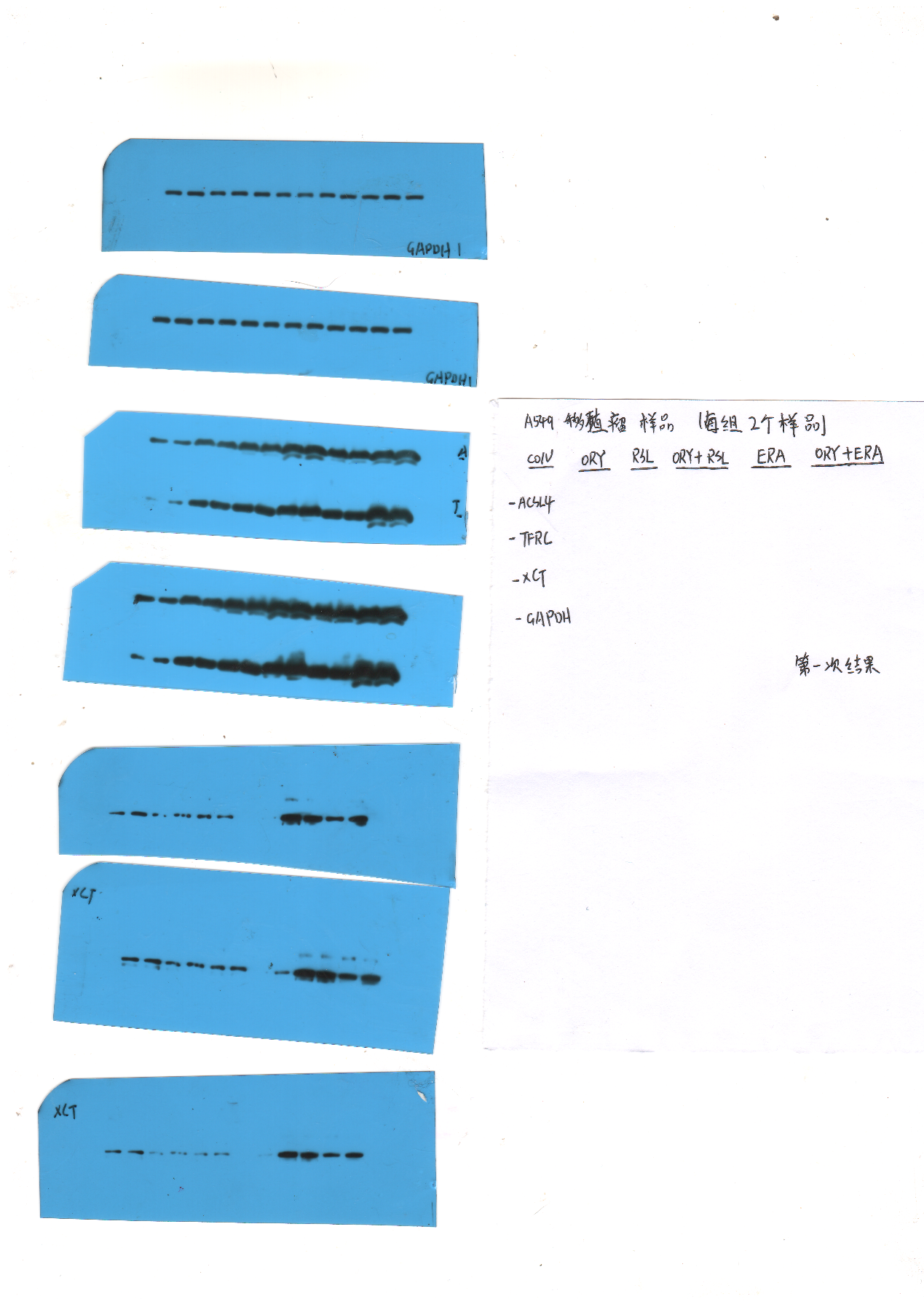


Supplementary Fig. 1H


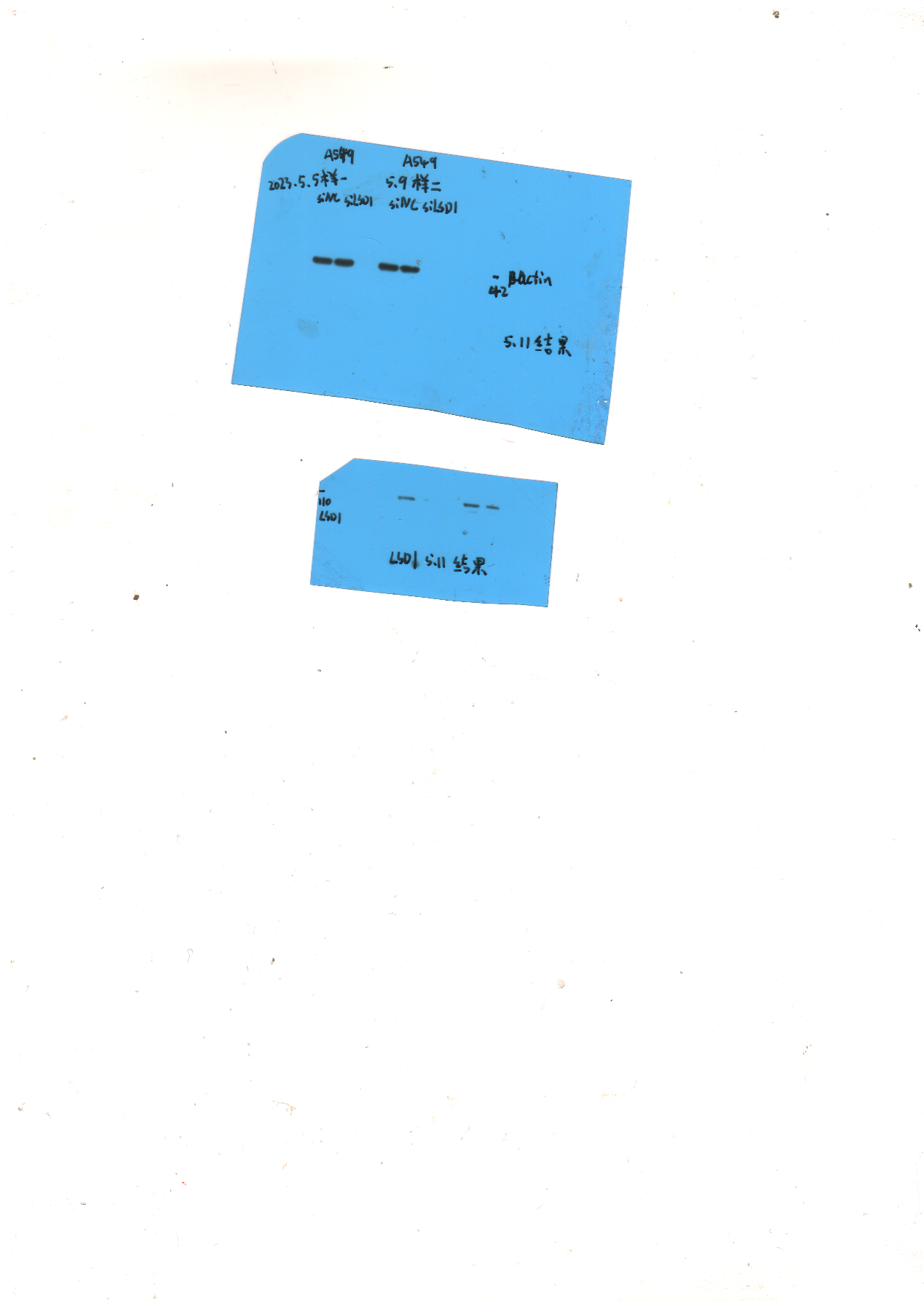


Supplementary Fig. 2A


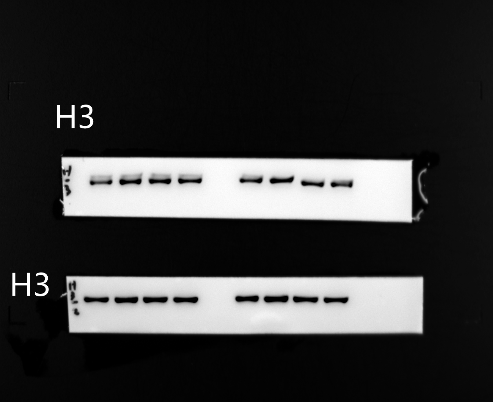

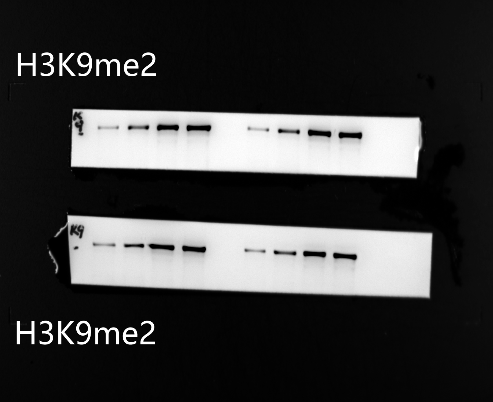


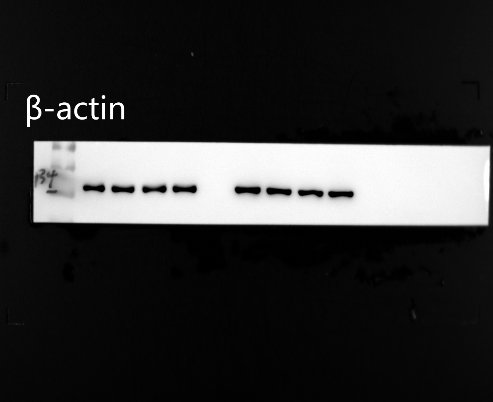

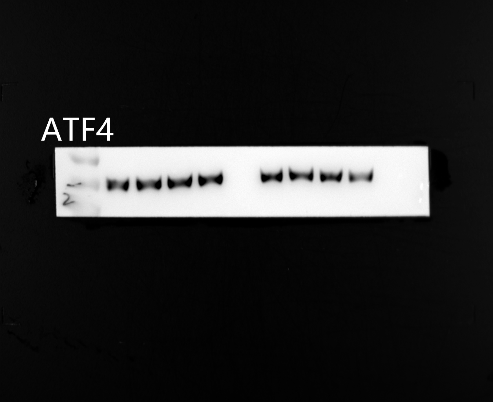


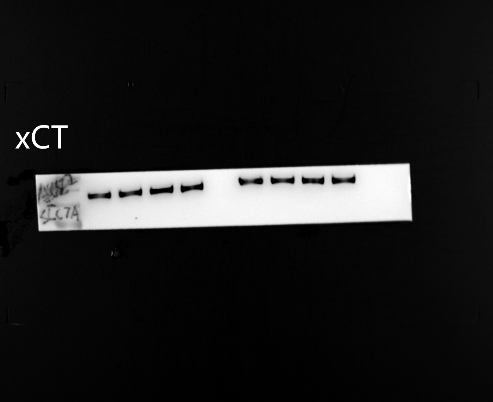


Supplementary Fig. 3A、4A


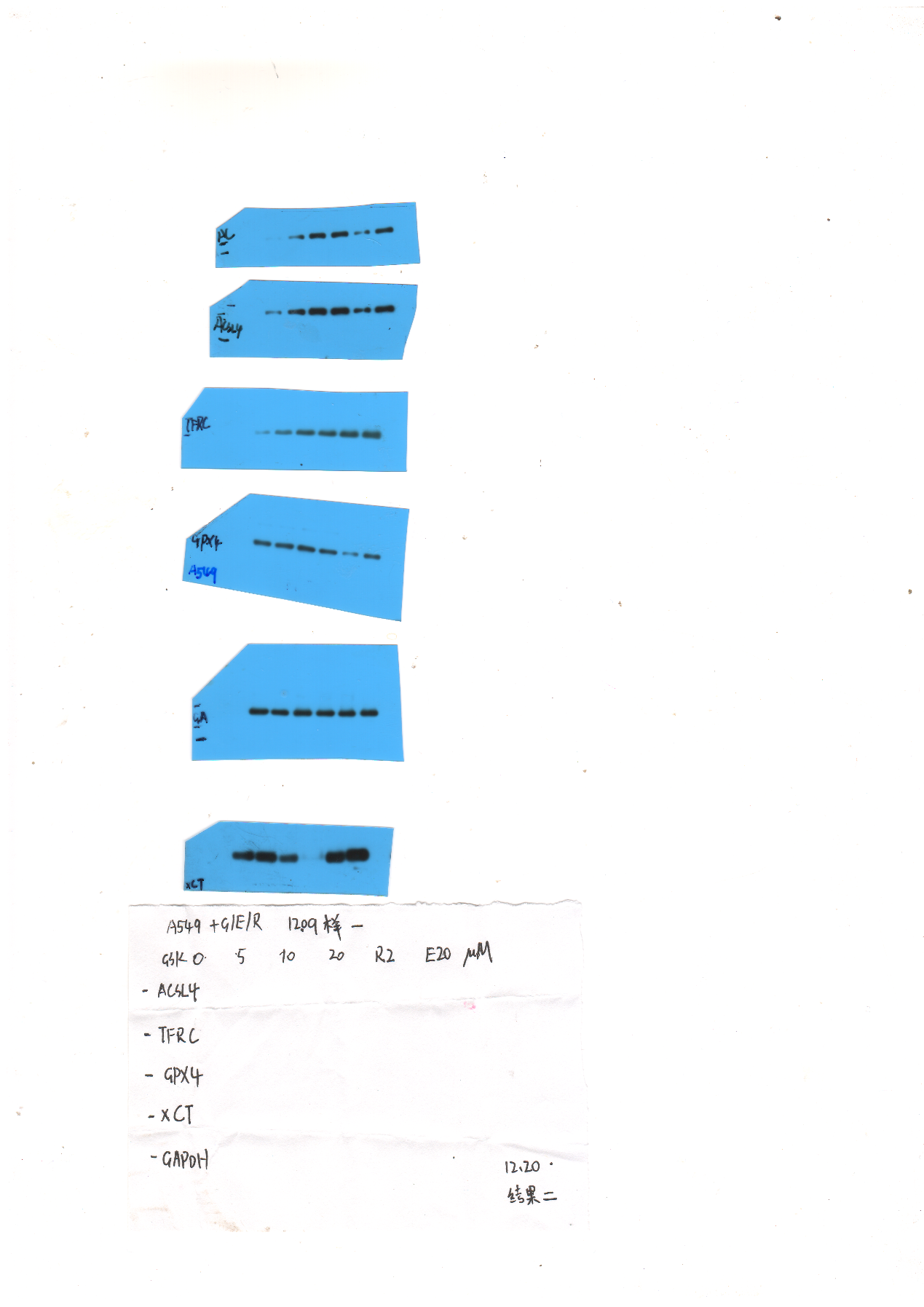


Supplementary Fig. 5B


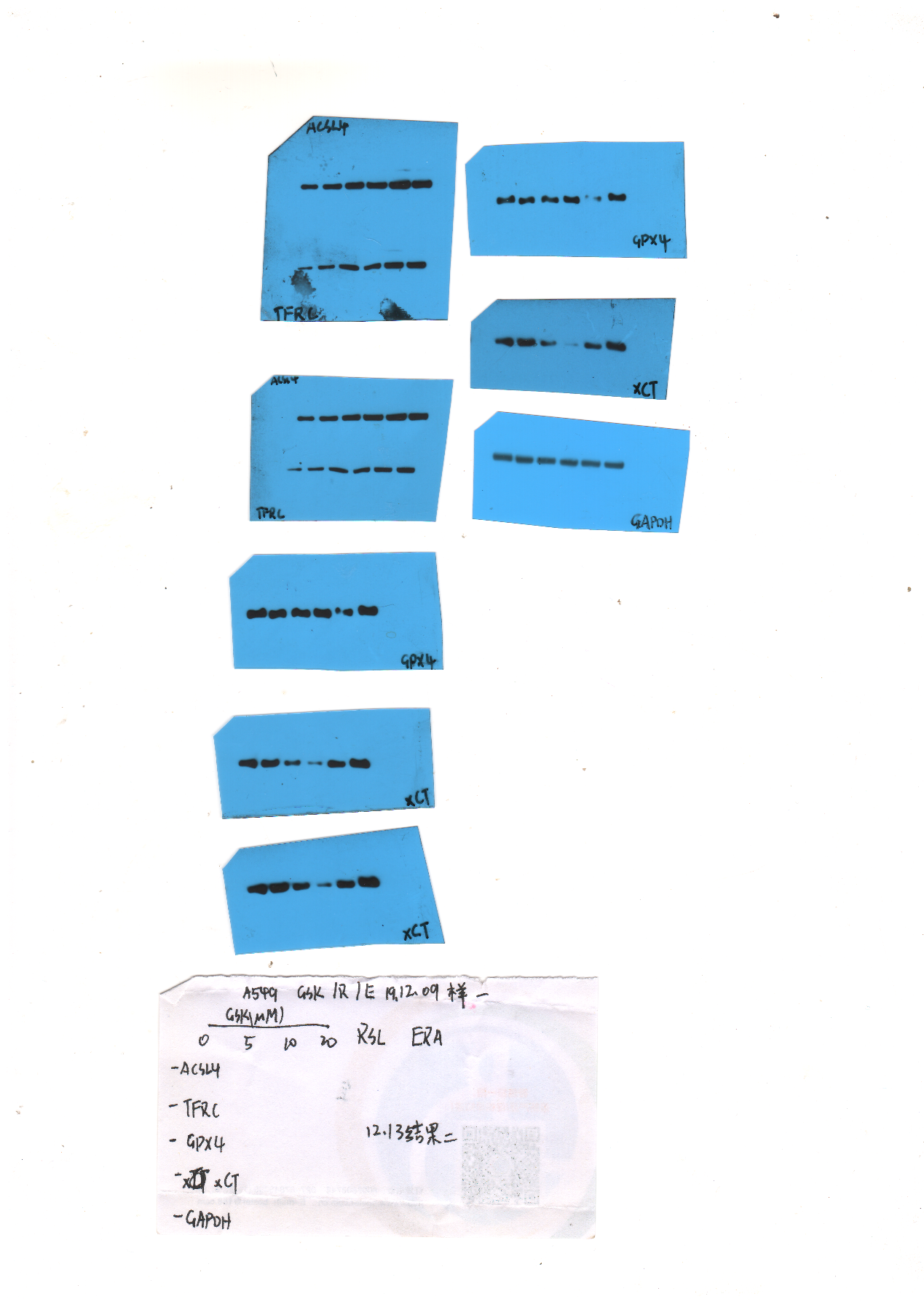


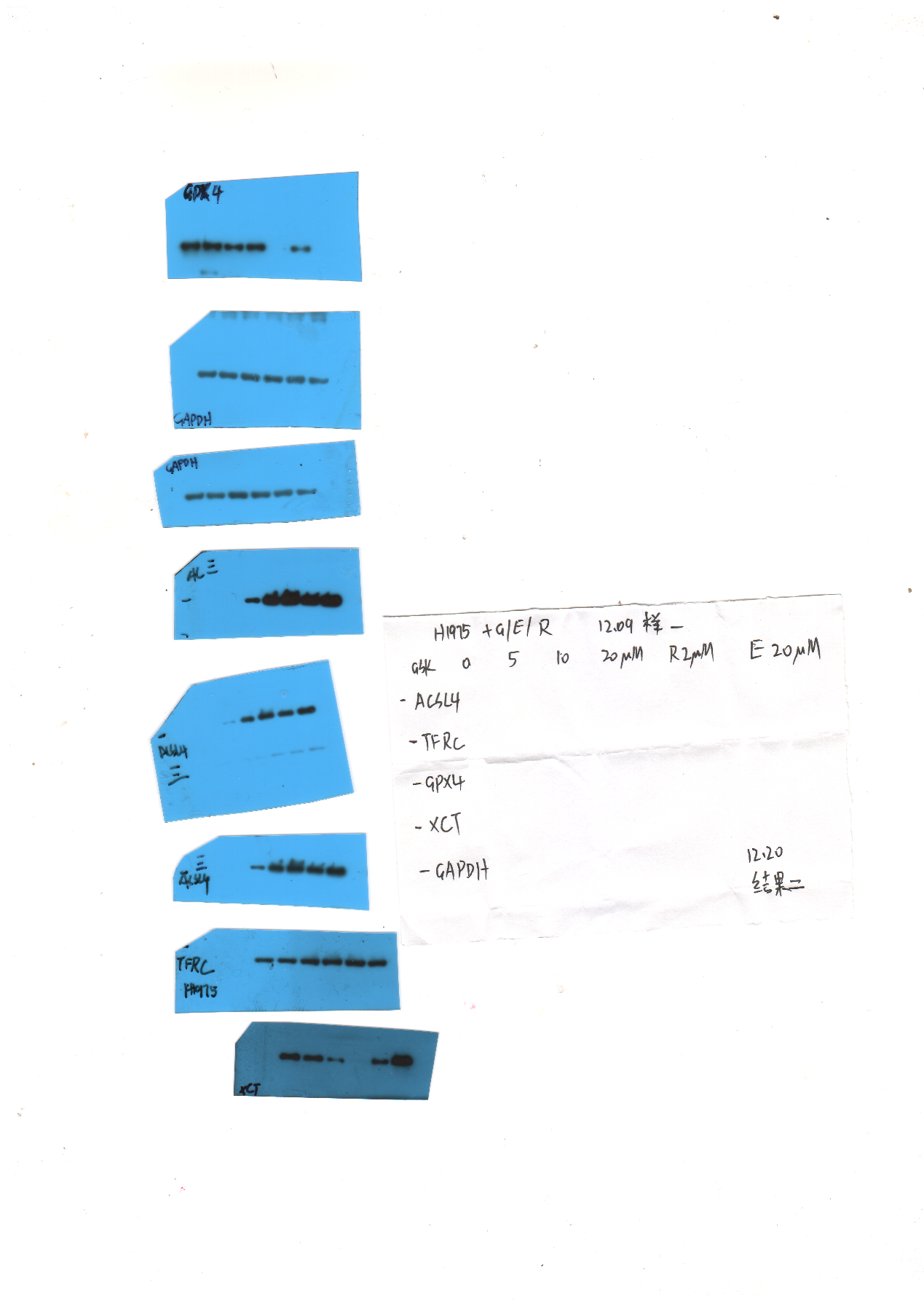


Supplementary Fig. 3E


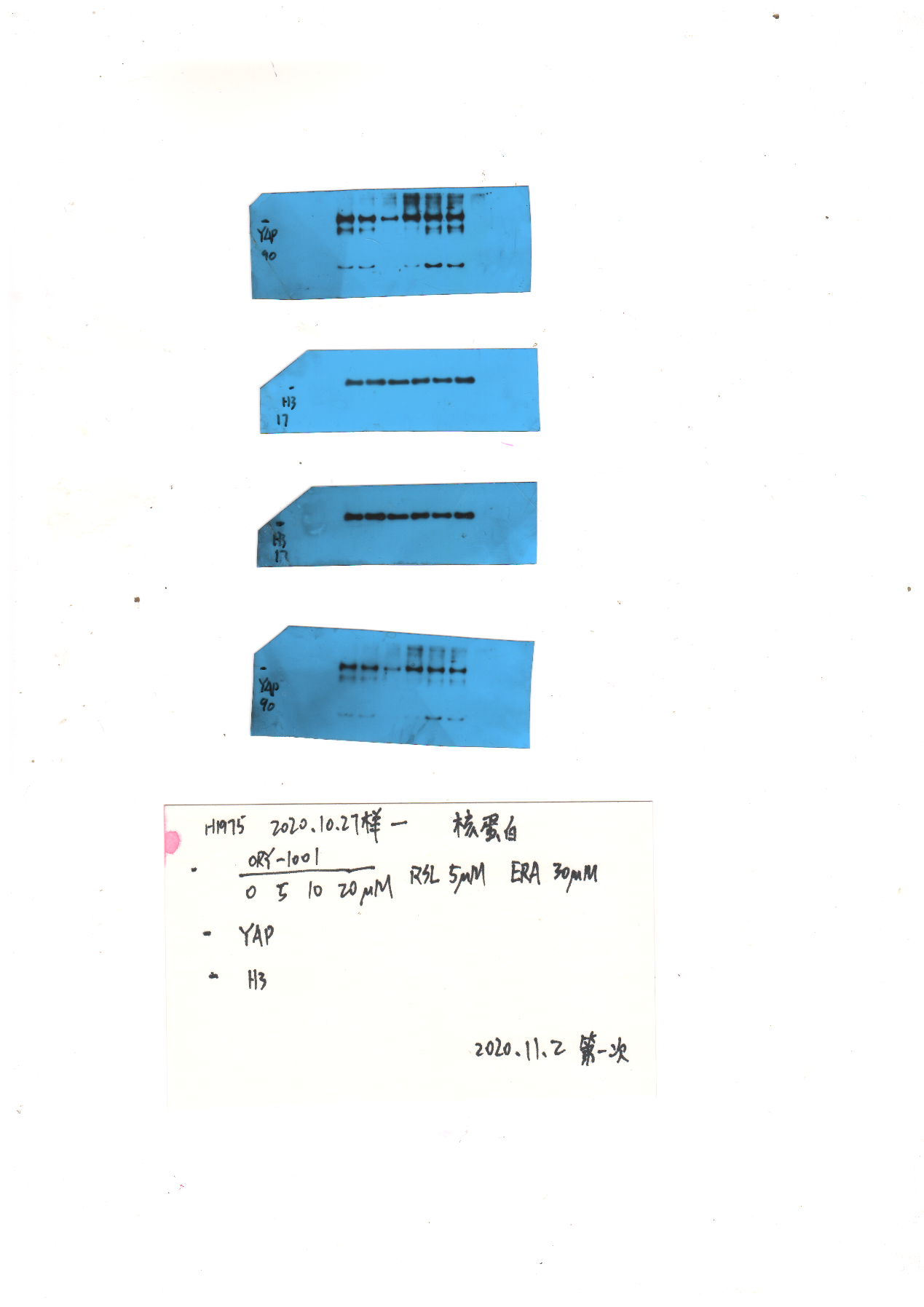


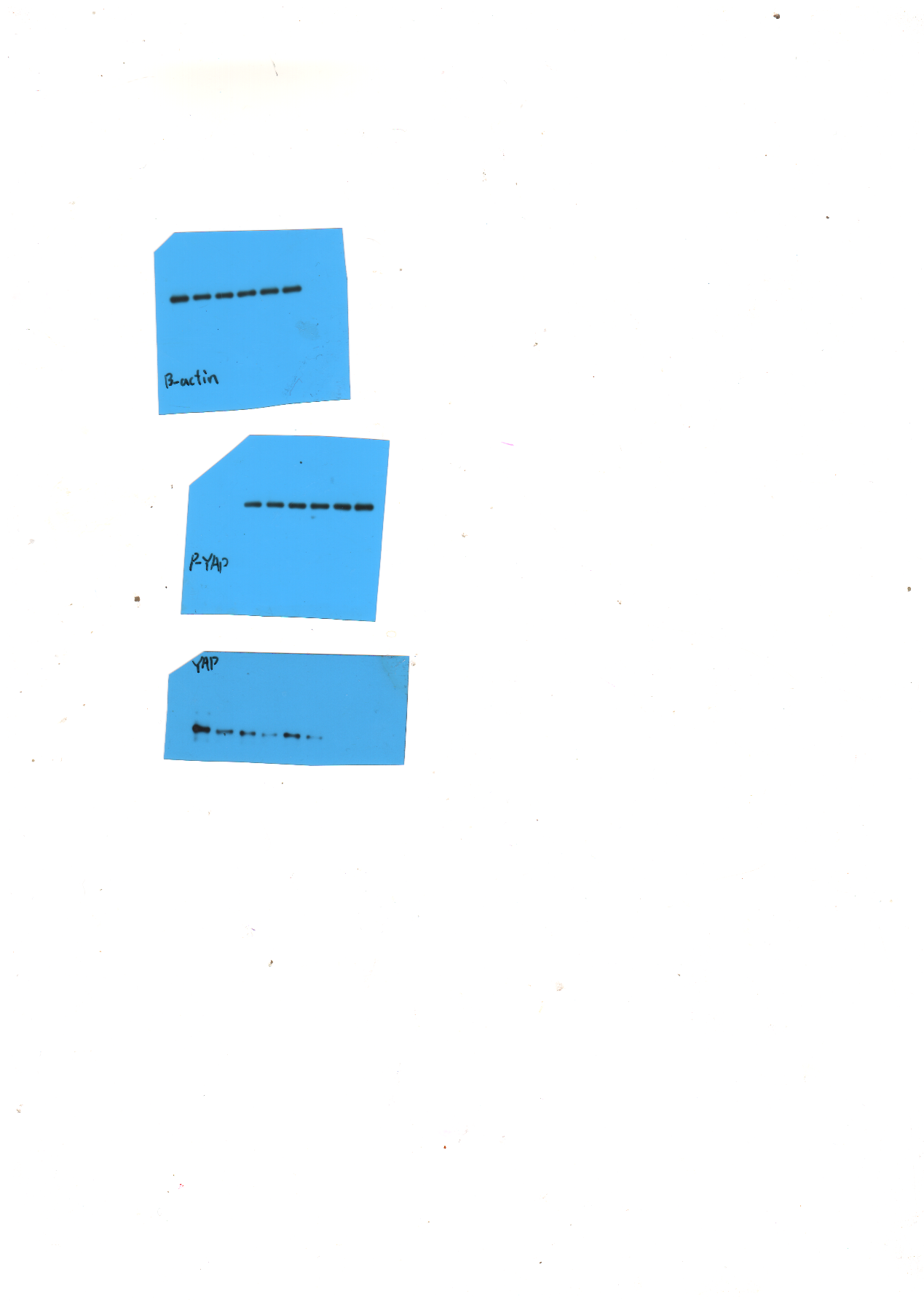


Supplementary Fig. 6A


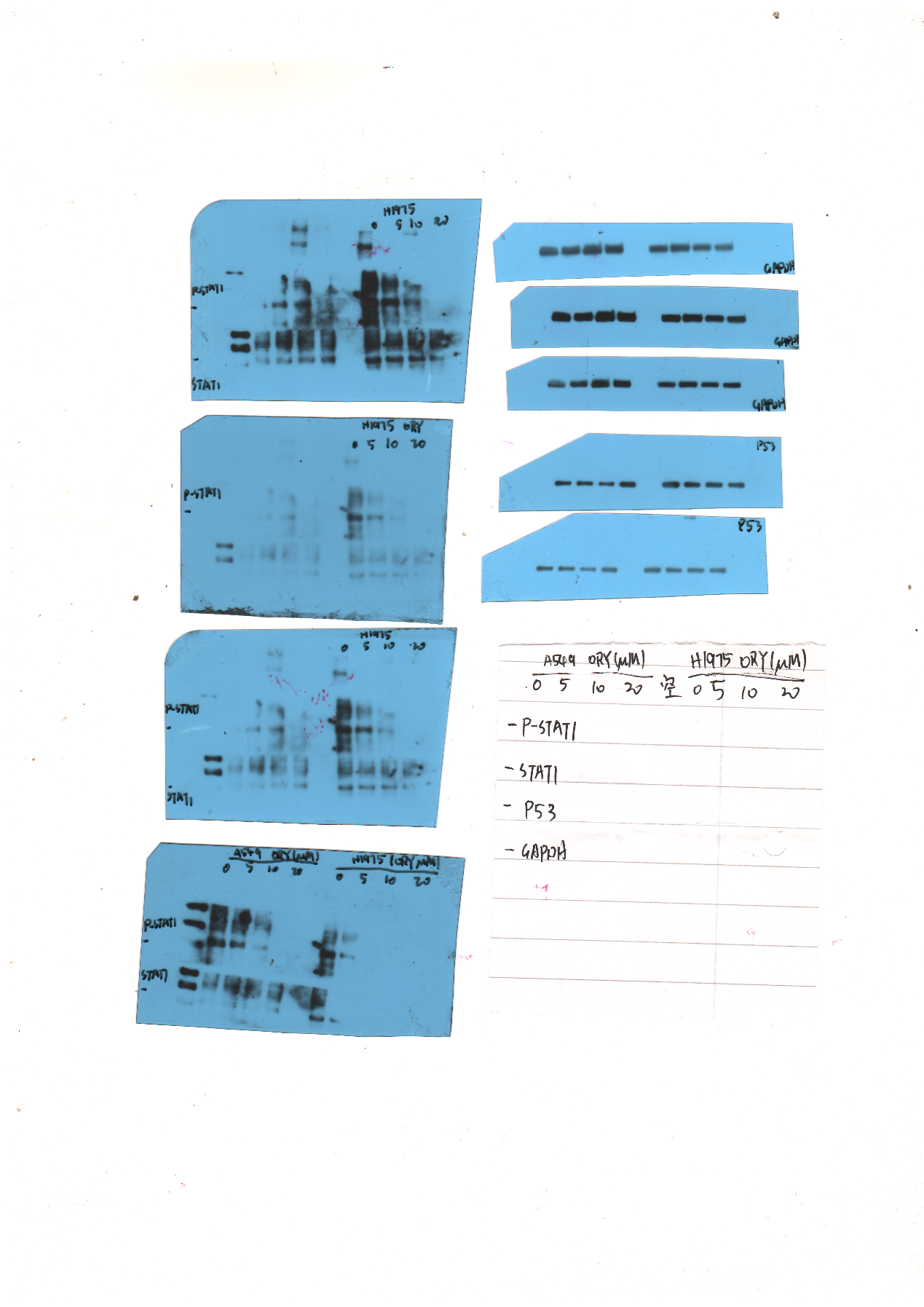


Supplementary Fig. 6C


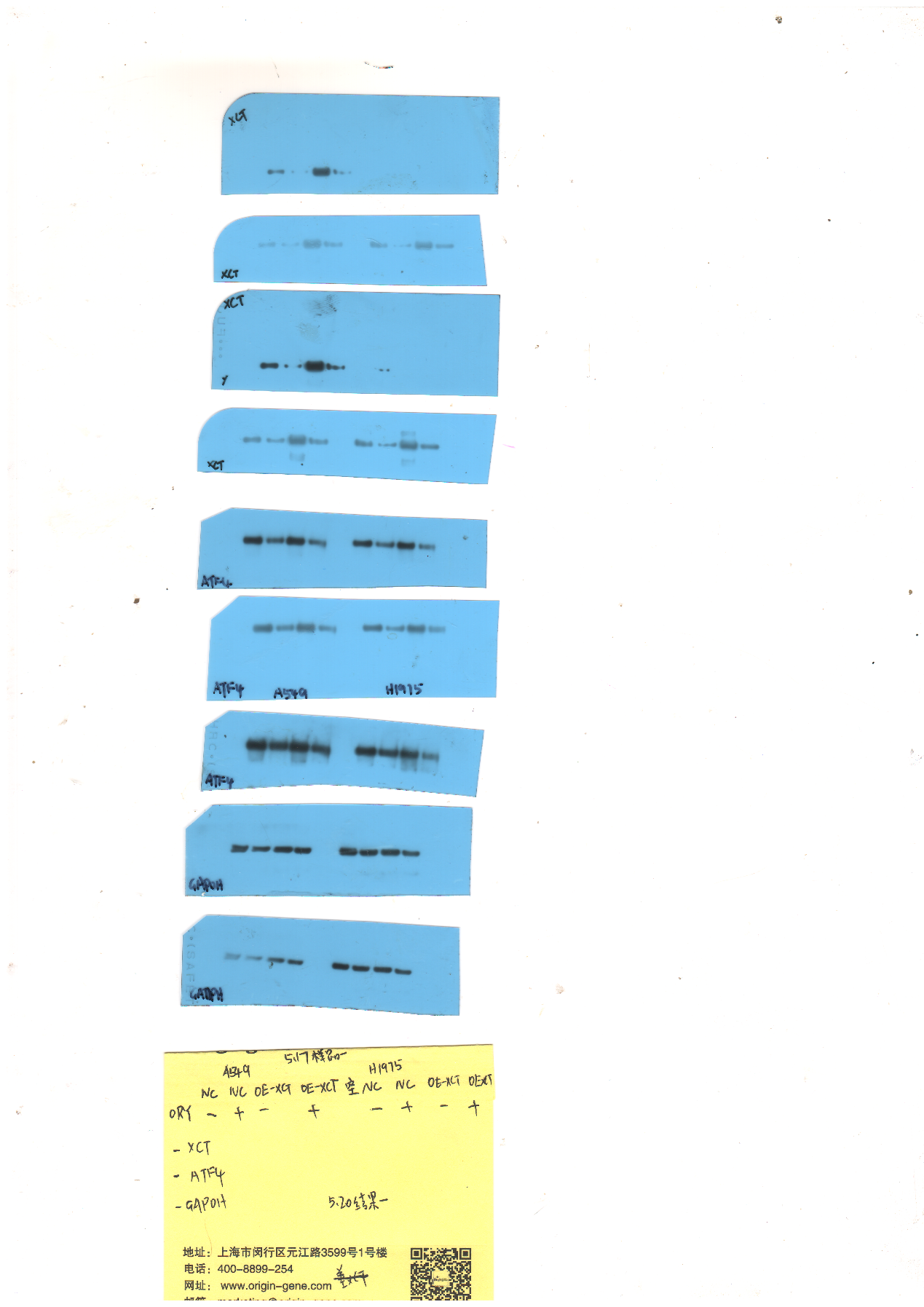


Supplementary Fig. 7F


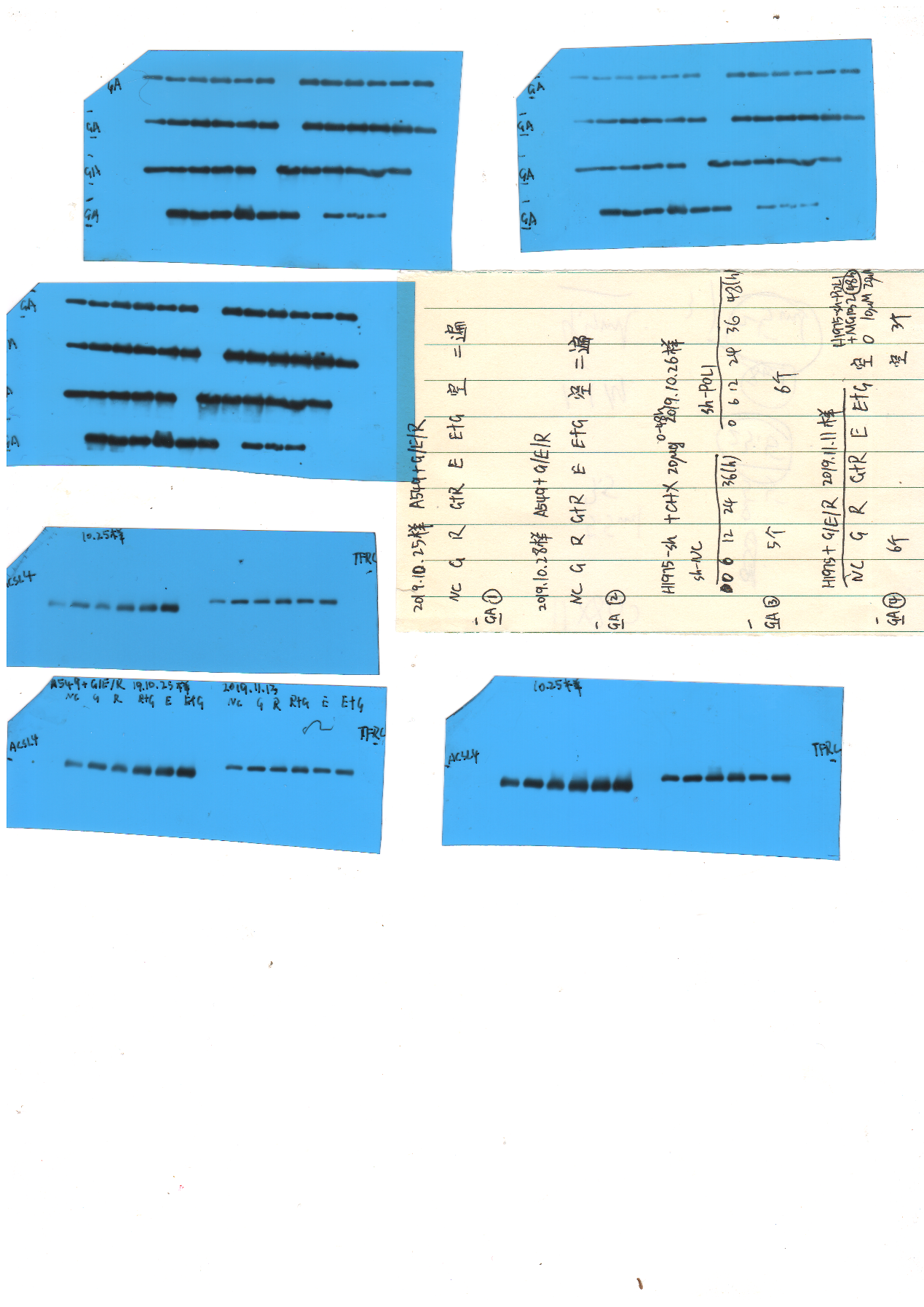


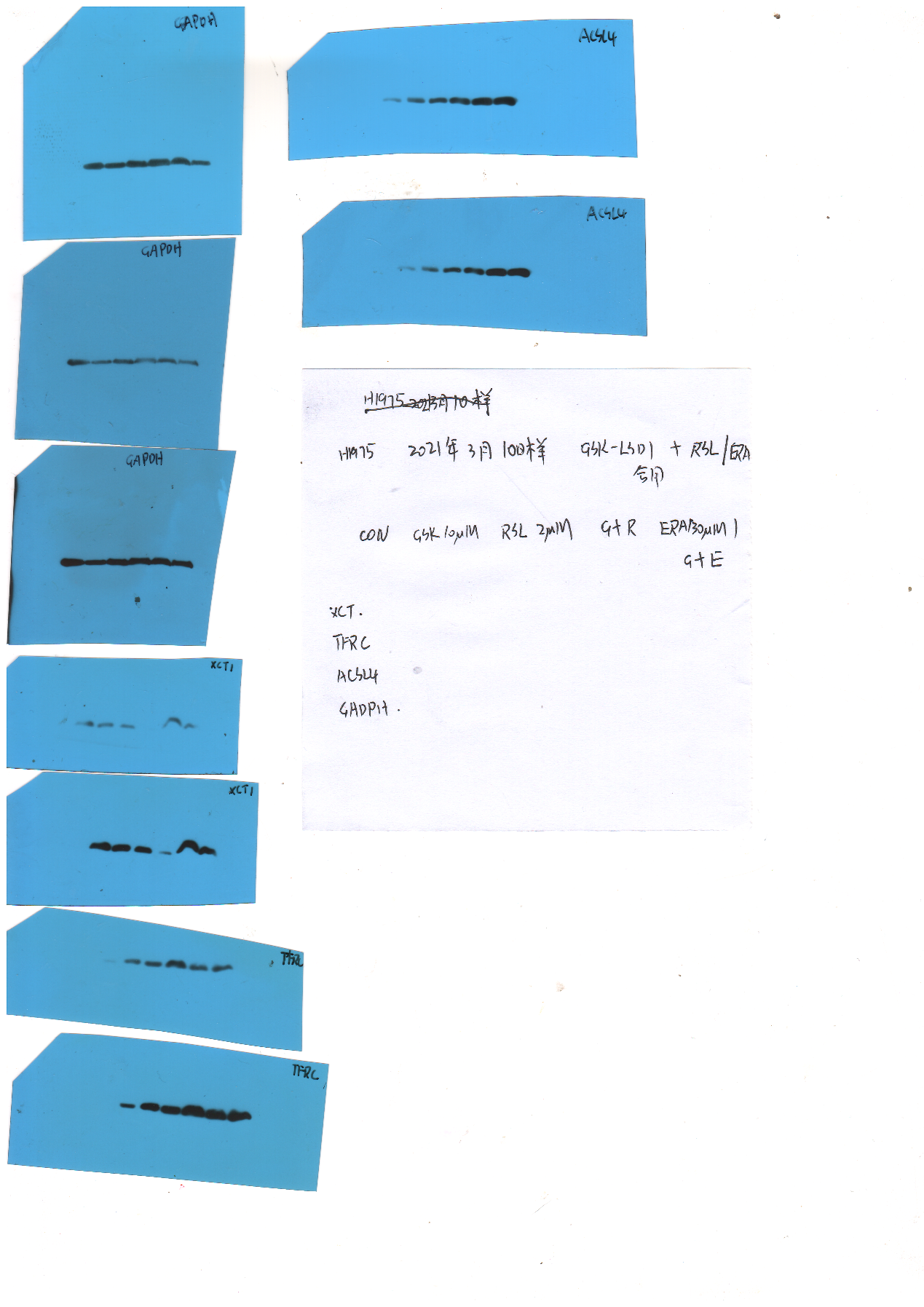

Supplement: Supplementary file 2 — Original Data File [file 41419_2023_6238_MOESM2_ESM.docx]
