# Supplementary material for: Inhibition of LSD1 induces ferroptosis through the ATF4-xCT pathway and shows enhanced anti-tumor effects with ferroptosis inducers in NSCLC
Source: Cell Death Dis. 2023 Nov 3;14(11):716. doi: 10.1038/s41419-023-06238-5 (PMC10624898; doi:10.1038/s41419-023-06238-5)
Supplement: Supplementary file 1 — Supplementary Materials [file 41419_2023_6238_MOESM1_ESM.docx]

**Supplementary Fig.**

Supplementary Fig. 1 Inhibiting LSD1 induces ferroptosis.

(A) Cell viability was detected by SRB assay in NSCLC cell lines treated with GSK at increasing concentrations for 96 h. (B) The expression of LC3-II and PARP were determined by western blotting in A549 and H1975 cell lines treated with increasing concentrations of ORY for 96 h. (C) Apoptosis was analyzed by flow cytometry in A549 and H1975 cells treated with increasing concentrations of ORY for 96 h. (D) After A549 and H1975 cells were treated with 10 μM GSK for 48 h, 5 μM Fer-1, 5 μM ZVAD, 2 μM Nec1, or 10 nM BA1 were added as indicated for 48 h again, and cell viability was measured by SRB. (E-G) After A549 and H1975 cells were treated with 5 μM, 10 μM or 20 μM GSK for 96 h, 20 μM ERA for 48 h or 2 μM RSL for 48 h as indicated, Lipid peroxides (E), cell death (F) and total ROS accumulation (G) were analyzed by flow cytometry. (H-I) After LSD1 was transiently silenced by using siRNA in A549 cell lines and cells were treated with 10 μM ORY for 96 h, LSD1 expression (H) was determined by western blotting, cell viability (I) was detected by SRB. (J) After LSD1 was transiently silenced by using siRNA in A549 cell lines and cells were treated with 5 μM Fer-1, 5 μM ZVAD, 2 μM Nec1, or 10 nM BA1 were added as indicated for 48 h again, and cell viability was measured by SRB. (K) After LSD1 was transiently silenced by using siRNA in A549 cell lines and cells were treated with 10 μM ORY for 96 h and production of lipid peroxides was analyzed by flow cytometry. (L) After A549-KOLSD1 cell lines were transfected with pIRES2-EGFP-LSD1 plasmid and the expression of LSD1 was examined by western blotting. (M) After A549-KOLSD1-OELSD1 cells were treated with 10 μM ORY/GSK for 48 h, 5 μM Fer-1 was added as indicated for 48 h again, and cell viability was measured by SRB. n ≥ 3; Mean ± SEM; ^ns^P > 0.05; *P < 0.05; **P < 0.01; ***P < 0.001.

Supplementary Fig. 2. Inhibiting LSD1 does not induce ferroptosis in normal lung epithelial cells.

(A) Cell viability was detected by SRB assay in NSCLC cell lines treated with GSK or GSK at increasing concentrations for 96 h. (B) Lipid peroxides were measured using flow cytometry in NSCLC cells treated with 10 μM ORY or GSK for 96 h. (C) The expression of xCT, ATF4 and H3K9me2 was determined by western blotting in BEAS-2B cell lines treated with increasing concentrations of ORY or GSK for 96 h. n ≥ 3.

Supplementary Fig. 3 GSK induces ferroptosis by upregulating TFRC.

(A) TFRC mRNA expression was determined by qRT-PCR in A549 and H1975 cell lines treated with increasing concentrations of ORY for 96 h. (B) TFRC expression was determined by western blotting in A549 and H1975 cell lines treated with increasing concentrations of GSK for 96 h. (C) After LSD1 was transiently silenced by using siRNA in A549 cell lines and TFRC mRNA expression was determined by qRT-PCR. (D-E) After TFRC was transiently silenced by using siRNA in A549 and H1975 cell lines and cells were treated with 10 μM GSK for 96 h, cell viability (D) was detected by SRB and the production of lipid peroxides (E) was analyzed by flow cytometry. (F) SP1 mRNA was determined by qRT-PCR in A549 cell lines treated with increasing concentrations of ORY for 96 h. (G) YAP expression was determined by western blotting in A549 and H1975 cell lines treated with increasing concentrations of ORY for 96 h. n ≥ 3; Mean ± SEM; ^ns^P > 0.05; ***P < 0.001.

Supplementary Fig. 4 GSK induces ferroptosis through the upregulation of ACSL4.

(A) ACLS4 expression was determined by western blotting in A549 and H1975 cell lines treated with increasing concentrations of GSK for 96 h. (B) After LSD1 was transiently silenced by using siRNA in A549 cell lines and ACSL4 mRNA expression was determined by qRT-PCR. (C-D) After ACLS4 was transiently silenced by using siRNA in A549 and H1975 cell lines and cells were treated with 10 μM GSK for 96 h, cell viability (C) was detected by SRB and the production of lipid peroxides (D) was analyzed by flow cytometry. n ≥ 3; Mean ± SEM; *P < 0.05; **P < 0.01; ***P < 0.001.

Supplementary Fig. 5 Decreased xCT is critical for GSK to induce ferroptosis

(A) GSH content was determined in A549 and H1975 cell lines treated with increasing concentrations of GSK for 96 h. (B) xCT expression was determined by western blotting in A549 and H1975 cell lines treated with increasing concentrations of GSK for 96 h. (C) The xCT mRNA levels were determined by qRT-PCR in A549 and H1975 cell lines treated with 10 μM GSK for 96 h. (D) Quantitative ChIP studies were conducted to characterize the enrichment of H3K9me2 at promoters of *xCT* gene in A549 cells treated with 10 μM ORY for 96 h. n ≥ 3; Mean ± SEM; *P < 0.05; **P < 0.01; ***P < 0.001.

Supplementary Fig. 6 ORY decreased xCT independently of these regulators.

(A) The expression of P53, p-STAT3, STAT3 and NRF2 was determined by western blotting in A549 and H1975 cell lines treated with increasing concentrations of ORY for 96 h. (B) The ARNTL, ATF3 and BACH1 mRNA levels were determined by qRT-PCR in A549 cells treated with increasing concentrations of ORY for 96 h. (C) The expression of xCT and ATF4 were measured by western blotting in xCT-overexpressing cells treated with 10 μM ORY for 96 h. (D) Quantitative ChIP studies were conducted to characterize the enrichment of H3K4me2 at promoters of ATF4 gene in A549 cells treated with 10 μM ORY for 96 h. n ≥ 3; Mean ± SEM; *P < 0.05; **P < 0.01; ***P < 0.001.

Supplementary Fig. 7 LSD1 inhibition sensitizes cancer cells to ferroptosis.

A549 and H1975 cells were treated with 10 μM ORY for 96 h and 2 μM RSL for 48 h alone or in combination as indicated. Cell viability (A) was detected by SRB. Production of lipid peroxides (B), cell death (C) and total ROS accumulation (D) was analyzed by flow cytometry, the levels of GSH (E) were detected by Total Glutathione Assay Kit and the expression of TFRC, ACSL4 xCT and ATF4 (F) was analyzed by Western blotting. n ≥ 3; Mean ± SEM; *P < 0.05; **P < 0.01; ***P < 0.001.

Supplementary Table

Supplementary Table 1. List of primer sequences for tilting ChIP at gene promoters

| **Genes** | **Primer** | **Sequence** | **Position (to TSS)** |
| --- | --- | --- | --- |
|  | P1-F | 5’-CCGGCTCCTTTCCAGAGTTTA-3’ | -1000bp |
|  | P1-R | 5’-TTCATAGCCGACACGGCG-3’ | -760bp |
|  | P2-F | 5’-ACTCAGGCTTGGAACAGGTAAC-3’ | -790bp |
|  | P2-R | 5’-CAGCCTGCGCGTCCTTC-3’ | -650bp |
|  | P3-F | 5’-CAGTGCGGACCTCCACTGA-3’ | -636bp |
|  | P3-R | 5’-CTCGGGTCGCTGCTAGTC-3’ | -440bp |
| ATF4 | P4-F | 5’-AAACTCTTCGCCGGAAAACG-3’ | -446bp |
|  | P4-R | 5’-AGTCCGTTCTTTCCTTCGCC-3’ | -160bp |
|  | P5-F | 5’-CCTTTTCTCGTCACAGCTACG-3’ | -171bp |
|  | P5-R | 5’-TCTCTATGGGGACGCAGCA-3’ | +260p |
|  | P6-F | 5’-CCTTTTCTCGTCACAGCTACG-3’ | +266bp |
|  | P6-R | 5’-TCTCTATGGGGACGCAGCA-3’ | +401bp |
|  | P1-F | 5′-TACCATCAGGAACAGGAAGCTAGT-3’ | -1000bp |
|  | P1-R | 5′-CAGCGAACTATCTGAGGGCTTC-3’ | -800bp |
|  | P2-F | 5′-AACAGCATTCGTATGACCGGC-3’ | -806bp |
|  | P2-R | 5′-AGGACGCAGGATTTTCACGTT-3’ | -600bp |
|  | P3-F | 5′-AGCGGGGATCTTAACGCAGT-3’ | -606bp |
| ACSL4 | P3-R | 5′-AAGTTCCCCTCGGGTCATCT-3’ | -400bp |
|  | P4-F | 5′-CGCAAGGACATCCCGAAACA-3' | -410bp |
|  | P4-R | 5′-ACGTCAGTCGAAAAGGCGGA-3' | -150bp |
|  | P5-F | 5′-CAGCGATTCGGCTGGCTC-3' | -195bp |
|  | P5-R | 5′-TTGGCAGAAAAAGCCGCAG-3' | +12bp |
|  | P6-F | 5′-GCATTAAGCAGTTAAACCGGC-3' | +0bp |
|  | P6-R | 5′-ATGTTTTGTCCCAAAGTATCAGTGT-3’ | +250bp |
|  | P1-F | 5’-GGGAGAGAGATTTTAGAGAATGGGA-3’ | -1000bp |
|  | P1-R | 5’-TCTGGGCCTTGGGCTTTTAC-3’ | -800bp |
|  | P2-F | 5’-GCTACTACCTCCCTGGCCT-3’ | -803bp |
|  | P2-R | 5’-TGCCATCGCTGTTATTGGATTG-3’ | -562bp |
| TFRC | P3-F | 5’-ATTACGCAAAGCACTCCGCT-3’ | -588bp |
|  | P3-R | 5’-ATGTTGGATATTGCACTGCGG-3’ | -348bp |
|  | P4-F | 5’-TGCAATATCCAACATTTAAGCGTCA-3’ | -374bp |
|  | P4-R | 5’-TCCTCCACCCTGGTAGCC-3’ | -104bp |
|  | P5-F | 5’-GCCTTTTCCCTTGGCCTT-3’ | -127bp |
|  | P5-R | 5ʹ-TGACAACCACTTCCTGAGGC-3ʹ | +120bp |

TSS, transcription start site

Supplementary Table 2. The sequences of the primers for the qRT-PCR

| **Genes** | **Forward Prime** | **Reverse Prime** |
| --- | --- | --- |
| ACSL4 | TCTGCTTCTGCTGCCCAATT | CGCCTTCTTGCCAGTCTTTT |
| ARNTL | GGAGCGGCTCATAGATGCAA | AACGTCGTGCTCCAGAACAT |
| ATF3 | CAGGTCTCTGCCTCGGAAGT | AGGGCGTCAGGTTAGCAAAA |
| ATF4 | CTTGATGTCCCCCTTCGACC | CTTGTCGCTGGAGAACCCAT |
| BACH1 | AGATGTGCTGTGCGATGTCA | CATCAGCCTGGCCTACGATT |
| PTGS2 | CTGGCGCTCAGCCATACAG | CGCACTTATACTGGTCAAATCCC |
| SP1 | GAGGGCAGGGTGCCAATG | CCACCAGAGACTGTGCGATT |
| TFRC | ACTTGCCCAGATGTTCTCAG | GTATCCCTCTAGCCATTCAGTG |
| xCT | GGTCCATTACCAGCTTTTGTACG | AATGTAGCGTCCAAATGCCAG |
| β-actin | GGGAAATCGTGCGTGACATT | GGAACCGCTCATTGCCAAT |

Supplementary Table 3. Cell Lines, Mutation Types, and Sensitivity to LSD1 Inhibitors

| Cell Line | Mutation Types | | | Sensitivity |
| --- | --- | --- | --- | --- |
|  | EGFR | KRAS | P53 |  |
| A549 | WT | Mut | WT | Yes |
| H1975 | Mut | WT | Mut | Yes |
| H1299 | WT | WT | Nul | NO |
| H460 | WT | Mut | WT | NO |
| H1650 | Mut | WT | Mut | NO |
| PC9 | Mut | WT | Mut | NO |

WT: Wild-type; Mut: Mutation; Nul: Null mutation.
